# Electrochemical behavior and theoretical studies of arylazo (1-naphthyl-2-cyanoacetamide) derivatives as new corrosion inhibitors for Inconel 800 in chloride solution

**DOI:** 10.1038/s41598-024-62795-5

**Published:** 2024-06-26

**Authors:** Mariem M. Motawea

**Affiliations:** 1 Department of Chemistry, College of Science, University of Bisha, Bisha, 61922 P.O. Box. 551, Saudi Arabia; 2 Department of Basic Science, Delta Higher Institute for Engineering and Technology, Mansoura, 35681 Egypt

**Keywords:** 1-Naphthyl-2-cyanoacetamide, Corrosion, Inhibition efficiency, Inconel 800, Langmuir adsorption, Activation parameters, Monte Carlo simulation, Chemistry, Materials science

## Abstract

In this study, synthesize and insight the corrosion inhibition properties of two novel derivatives of 1-naphthyl-2-cyanoacetamide (NCDs) [2-cyano-2-((5,6-dimethyl-1H-benzo[d]imidazol-2-yl) diazenyl)-*N* (naphthalene-1-yl)acetamide] (NCD1) and [2-Cyano-*N*-(naphthalene-1-yl)-2-[(4,6-dimethyl-1H-pyrazolo [3, 4-b] pyridine-3-yl) hydrazono] acetamide] (NCD2). The characterization of the synthesized NCDs was confirmed through the utilization of Mass fragmentation analysis, ^1^H-NMR, and IR. The corrosion inhibition performance of NCDs as a novel and environmentally safe corrosion inhibitor has been investigated by electrochemical techniques, a chemical technique, and theoretical studies for its anti-corrosion behavior of Inconel 800 in chloride medium. In addition, the surface morphology and inhibitor adsorption on the Inconel 800 surface were confirmed utilizing SEM, EDX, FTIR, and AFM. The advantages of NCDs include their low toxicity, environmental friendliness, ease of preparation, low odor, contain (N, O, and π-Bonds), and the inhibition efficiency elevated with decreasing solution temperature as well as inhibitor dose increase, yielding increased efficiencies of 91.8% and 95.7% for NCD1 and NCD2, respectively, at the optimum concentration of 21 $\times$ 10^–6^ mol. L^−1^ and 298 K temperature. An analysis of Tafel plots reveals that NCDs adhere to a mixed and isothermal Langmuir adsorption mechanism. Density Functional Theory (DFT) and Monte Carlo (MC) simulation manifest the two compounds of NCDs can be adsorbed at the Fe (110) surface in a paralleled way, and can have a smaller energy gap (ΔE) value and exhibit higher efficiency. The experimental and theoretical findings confirm that the synthesized compounds obtained are capable of protecting the Inconel 800 from corrosion by creating an anti-corrosion coating on the surface.

## Introduction

Stainless steel is extensively utilized in petroleum facilities, including heat exchangers, storage tanks, and pipelines. However, it is susceptible to corrosion when exposed to acids like HCl, commonly employed to eliminate scaling or rust^1,2^. According to the World Corrosion Organization, recent surveys indicate that the global direct corrosion cost ranges from 1.3 to 1.4 trillion €, equivalent to (3.1–3.5%) of a country's annual GDP. Corrosion is a natural substance deterioration due to its spontaneous interaction with the environment^3^. Utilizing organic heterocyclic compounds as corrosion inhibitors is common because heteroatoms are capable of donating lone pairs of electrons^4^. These compounds, incorporating electron-donating heteroatoms like sulfur, nitrogen, and oxygen, along with π-electrons and donor groups like OH, NH_2_, and CH_3_, are frequently utilized in industry to inhibit corrosion and form protective films on exposed surfaces^5–7^. Heterocyclic compounds offer the metal surface unoccupied orbitals, such as iron, to attract and facilitate the adsorption process by providing complementary electrons. As a result, the metal reverts to its stable condition and prevents oxidation^8,9^, preserving the material and extending its lifespan^10^. The selection of an inhibitor is determined by its efficacy, environmental side effects, and cost-effectiveness^11,12^. Notably, the compounds under investigation possess key attributes that render them highly suitable for use as effective inhibitors. Specifically, their composition includes both sulfur and nitrogen atoms, besides aromatic rings. These features contribute to enhanced adsorption of the compound onto the surface of steel, thereby more efficient inhibition. In addition, the compounds are readily synthesized, inexpensive, environmentally friendly materials that readily mix with acidic solutions^13,14^. This is confirmed by unoccupied d-orbital occurrence in iron atoms, which establish coordinative bonds with atoms capable of donating electrons. Furthermore, there is interaction between rings that possess conjugated π-electrons. Pyrimidines containing heterocycles have been proven to be efficient and secure inhibitors that exhibit remarkable corrosion inhibition properties (in acidic media) on copper metal^15^. Cyanoacetamide and its derivatives have been reported as powerful and eco-friendly inhibitors because of their containing N and O heteroatoms^16^

This study examined the corrosion inhibition effects of two NCDs compounds. The results demonstrate that these derivatives effectively function as corrosion inhibitors. Therefore, it is advisable to conduct additional research in this area. Moreover, given the current environmental problems, these substances have non-toxic appearances, in addition to being easily dissolved in the test solution, thereby enhancing their effectiveness in providing protection. Several organic compounds containing "N" have been commonly referred to in the literature as corrosion inhibitors in steel (utilizing H_2_SO_4_ and HCl solutions). The predicted percentage of their inhibition efficiency is depicted in Table 1.

**Table 1:** Performance comparison of organic derivatives containing nitrogen atoms utilized as corrosion inhibitors for steel and in this study.

Organic derivatives	Sample	Medium	%IE	Ref
4-(5-Benzoyl-2-imino-6-phenyl-1,2,3,4-tetrahy dropyrimidin-4-yl)benzoic acid	SS	1 M HCl	90 at 5 × 10^−3^ M	^17^
5-((2-Hydroxyethoxy)methyl)-2-methylquinolin-8-ol	Mild steel	1 M HCl	88.8 at 10^–3^ M	^18^
(E)-2-(1-(Pyridin-2-yl)ethylidene) hydrazine carboxamide	Mild steel	1 M HCl	77.7 at 10^–3^ M	^19^
1-(3-(4-Methoxyphenyl)-5-(quinoxalin-6-yl)-4,5-dihydro-1H-pyrazol-1-yl)butan-1-one	Mild steel	1 M HCl	68 at 100 ppm	^20^
2-Amino-6-ethyl-5-methyl-[1,2,4]triazolo[1,5-a]pyrimidin-7-yl acetate	mild steel	1M HCl	84, at 10^–3^ M	^21^
3-(2-(4-(Hydroxymethyl)-1H-1,2,3-triazol-1-yl) ethyl)-2-methyl-6,7,8,9-tetrahydro-4H-pyrido[1,2-a]pyrimidin-4-one	Mild steel	1 M HCl	91 at 5 mM	^22^
(E)-3-(8-(trifluoromethyl)quinolin-4-yl)-*N*'-(2,3,4-trihydroxybenzylidene)propanehydrazide	Mild steel	1 M HCl	88.9 at 11 × 10^–4^ M	^23^
(E)-*N*'-(4,6-dimethyl-1H-pyrazolo[3,4-b]pyridin-3-yl)-2-(naphthalen-1-ylamino)-2 oxoacetohydrazonoyl cyanide	Inconel	2 M HCl	95.7 at 21 × 10^–3^ M	Present work

After careful analysis of a wide range of literature (represented in Table 1) for comparison, it was observed that the molecules with more than one hetero atom (nitrogen (N), sulphur (S), oxygen (O)) and aromatic rings have high inhibitory efficiency^24^. One of the most widely used, effective, and useful corrosion inhibitors is organic molecules. The advantages of NCDs include their low toxicity, environmental friendliness, ease of preparation, low odor, lack of corrosive bleaching effect, and contain (N, O, and π-Bonds). It is also compatible with lower concentrations, which helps reduce costs while maintaining adequate protection.

In this study, we synthesized two novel derivatives of NCDs. We then investigated Inconel 800 corrosion inhibition behavior in a 2.0 M HCl solution using various techniques involving electrochemical impedance spectroscopic (EIS) analysis and potentiodynamic polarization (PDP). Advanced techniques, such as SEM, EDX, FTIR, and AFM were utilized to analyze the surface morphology. Furthermore, Monte Carlo simulation and quantum chemical calculations were employed.

## Experimental materials and techniques

### Materials

Inconel 800 specimen analysis indicated the presence of the following components: 46.1% Fe, 0.99% Mn, 0.17% Co, 0.14% Mo, 0.12% P, 0.91% Si, 0.49% Ti, 19.63% Cr, and 30.39% Ni. For WL tests, the Inconel 800 samples' geometric scale was 20 × 20 × 2 mm, while for electrochemical measurements, the exposed surface area was 10 × 10 mm, additionally, samples were abraded before the assessment (with varying emery papers’ grades, 320–2500) until a mirror finish appeared.

### Inhibitors

**Scheme 1:**
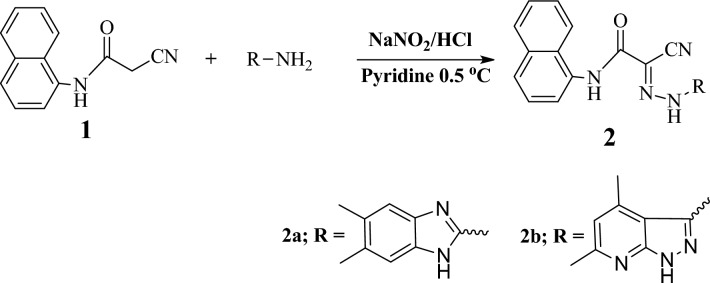
Coupling reactions of compound 1 with different heteroaromatic amines.

The 1-naphthyl-2-cyanoacetamide (1) was reacted in pyridine with heteroaromatic diazonium salts to produce the corresponding coupling products (NCDs)^25^. The products (NCD1&2) were synthesized (following the detection of the CN group) at 2220 cm^-1^ in the IR spectra. Confirmation of synthesized inhibitors (NCD1) chemical structure: in 78% Brown powder yield; mp > 300 °C; “IR (KBr): υ/cm^-1^ = 3435, 3247, 3233 (3NH), 1687 (C = O). ^1^H NMR (400 MHz, DMSO-*d*_6_): δ/ppm = 2.17 (s, 3H, CH_3_), 2.27 (s, 3H, CH_3_), 6.70 (s, 1H, CH) 7.51–8.05 (m, 9H, Ar–H), 10.2 (s, 1H, NH) 10.3 (s, 1H, NH); ^13^C NMR (100 MHz, DMSO-*d*_*6*_): δ/ppm = 19.4, 26.3, 109.6, 116.2, 121.8, 122.6, 125.6, 125.9, 126.1, 126.2, 127.6, 127.7, 127.8, 128.2, 132.7, 133.7, 155.4, 162; Anal. Calcd. for C_22_H_18_N_6_O (382.15): C, 69.10; H, 4.74; N, 21.98%, Found: C, 69.18; H, 4.78; N, 21.93%”. NCD2: in 76% brown powder yield; mp 204–205 °C; “IR (KBr): υ/cm^−1^ = 3490 (NH), 2215 (CN), 1639 (C=O). ^1^H NMR (400 MHz, DMSO-*d*_6_): δ/ppm = 2.56 (s, 3H, CH_3_), 2.27 (s, 3H, CH_3_), 4.08 (s, 1H, CH), 7.49–8.09 (m, 9H, Ar–H), 10.27 (s, 1H, NH); MS *m/z* (%): 384 (M+, 0.9), 383 (0.2), 341.7 (58.9), 278 (3.8), 236 (3.8), 208 (3.1), 143 (100), 127 (4); Anal. Calcd. for C_21_H_17_N_7_O (383.42): C, 65.79; H, 4.47; N, 25.57%; Found: C, 65.85; H, 4.56; N, 25.49%”. Scheme 1 depicts two NCD1 and 2, whereas Table 2 lists molecular formulas and chemical structures. The required concentrations (5 × 10^–6^–21 × 10^–6^) were prepared by dilution of stock solutions of NCDs with doubly distilled water. The examinations were carried out at varying concentrations (21 × 10^−6^, 17 × 10^−6^, 13 × 10^−6^, 9 × 10^−6^, and 5 × 10^−6^ M) with and without inhibitors. Moreover, experiments were performed in thermostatic conditions.

**Table 2:**
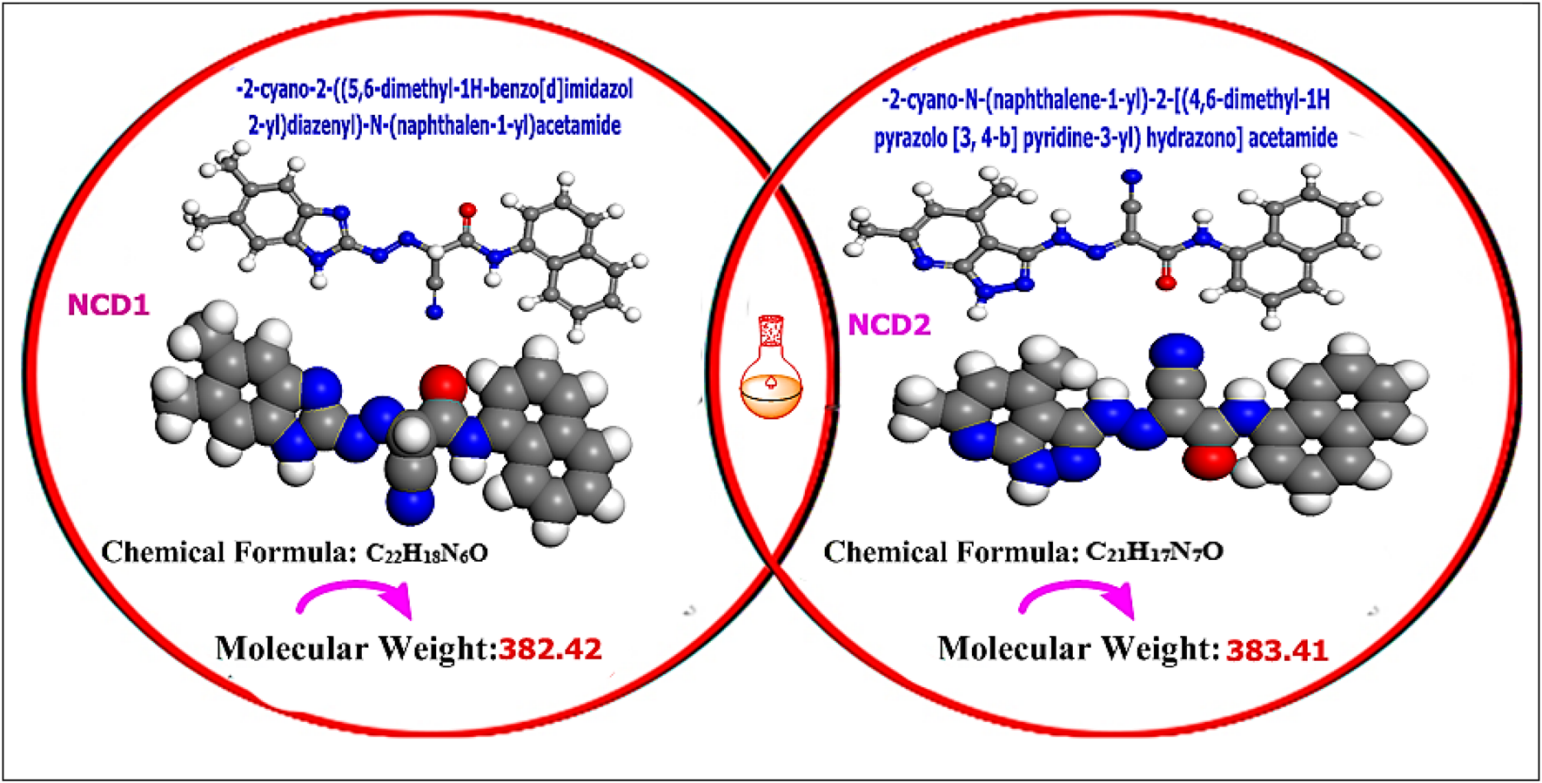
Chemical structure of NCDs.

### Solutions

A corrosive medium of 2.0 M HCl acid was created by dissolving concentrated 37% HCl (of research-grade) with distilled water. This medium was subsequently utilized to prepare five various molar concentrations (of each inhibitor: 5, 9, 13, 17, and 21 × 10^–6^ M).

### Electrochemistry tests

ZRA analyzer (Gamry reference: 3000 Potentiostat–Galvanostat) was utilized (in conjunction with 7.8.2 V of Gamry framework-data-acquisition-software). “The electrochemical analysis was carried out to examine the inhibition behavior (of newly synthesized inhibitors) against Inconel 800in 2.0 M HCl. Inconel 800 corrosion rate was assessed through the utilization of potentiodynamic polarization (PDP) and electrochemical impedance spectroscopy (EIS) techniques. The electrochemical measurements were conducted utilizing a cell comprising three distinct electrodes. The working electrode consists of a flat surface made of a Rod from Inconel 800 (1 cm^2^ area), securely held in a plastic holder (as the working electrode), saturated calomel electrode (SCE; as a reference electrode), and graphite electrode (CE; as a counter electrode). The data was fitted, graphed, and plotted using the 7.8.2 V of Gamry Echem Analyst software. Before commencing each measurement, the open circuit potential (OCP) was initially determined to achieve a stable state. EIS was conducted at 25 °C utilizing a low voltage (10 mV) as well as a 100 kHz to 20 MHz frequency range”. PDP was performed by automatically varying the potential from −1500 to 500 mV vs. SCE at the open circuit potential, using (1 mV s^−1^) scan rate at 25 °C.

### Chemical measurements (weight loss)

Weight loss tests were conducted following the standard procedure established by the American Society for Testing and Materials (ASTM) standard procedure^26^. “Weight measurements were performed utilizing five varying temperatures: 298, 308, 318, 328, and 338 k. The specimens were abraded utilizing emery paper of varying grades and subsequently washed with demineralized water followed by acetone. Finally, they were dried using air”. At temperatures ranging from 25 to 65 °C, the specimens, after weighting, were submerged in 100 ml beakers containing different concentrations of *NCDs* along with 2 M HCl. After varying times (1 to 6 h), every hour the Inconel 800 specimens were removed from the solution, washed, dried, and weighed once more. Determination of inhibition efficiency was done utilizing the equations as follows:

1 $$\theta = \frac{{\left( {W_{o} - W_{inh} } \right)}}{{W_{o} }}$$

2 $$\eta_{w} \% = \left[ {\frac{{\left( {W_{o} - W_{inh} } \right)}}{{W_{o} }}} \right] \times 100$$

where W_0_ is the weight loss value (without NCDs), θ is the surface coverage, and W_inh_ is the weight loss value (with NCDs).

The corrosion rate (C.R) (mg cm^−2^ min^−1^) is determined based on the formula as follows:

3 $$C.R = \frac{W}{{\left( {At} \right)}}$$

where A is the steel specimen surface area by cm^2^, W is the overall weight loss in mg, and t denotes the immersion time in minutes^27^.

### Surface morphology studies utilizing SEM, EDX, FT-IR, and AFM

#### Scanning *electron* microscopy (SEM) with energy dispersive X-ray analysis (EDX)

Surface analysis was conducted by using SEM–EDX JEOL-JSM-6510LV-Japan utilized for examining elemental composition as well as the surface morphology of corroded and glossy Inconel 800 (with and without the utilization of inhibitors) to examine the impact of inhibitors utilized on surface morphology as well as to demonstrate the protective properties of the treated surface. The samples were submerged (24 h) in HCl 2.0 M (with and without NCDs) at the optimal concentration. In addition, they were dehydrated at room temperature (prior to analysis). Also, the quantitative concentration of precipitated elements on the steel surface from the solutions is calculated using EDX.

#### Fourier-transform infrared spectroscopy (FT-IR)

The spectral resolution was set at 4 cm^−1^, and 128 scans were performed. The pure inhibitors were subjected to FT-IR analysis utilizing Attenuated Total Reflectance IR spectroscopy (ATR-IR) on the specimens obtained from the test solution after a 24-h immersion. This analysis aimed to compare and confirm inhibitors’ adsorption on the Inconel 800 surface utilizing Thermo Fisher Scientific-USA-Nicolet iS10.

#### Atomic force microscopy (AFM) tests

Prior to the examination, each Inconel 800 was submerged (in corrosive media) with and without one of the NCDs (for 24 h). The surface roughness parameters in both two-dimensional (2-D) and three-dimensional (3-D) microscales were subsequently determined utilizing AFM.

### Computational methods

#### Quantum chemical calculations

The synthesized inhibitors' geometrical ground state was optimized utilizing the density functional theory (DFT) method with Beck's three-parameter exchange function with basis sets 6-311G and Lee–Yang–Parr correlation function (B3LYP). Utilizing *ω*, σ, Δ*E*_back-donation_, dipole moment (*μ*) and Accelrys Material Studio can be expressed as energy values for LUMO and HOMO. Based on Koopman's theory^28^, A can be estimated from E_LUMO_^29^, and I can be determined (from E_HOMO_) utilizing the equations as follows:

4 $$A\left( {Electron \, affinity} \right) = - E_{LUMO}$$

5 $$I\left( {Ionization \, potential} \right) = - E_{HOMO}$$

Electronegativity (χ) and Global hardness (η) can be quantified utilizing the following equations^30^ according to (A) and (I) values.

6 $$\eta = {{\left( {{\text{I}} - {\text{A}}} \right)} \mathord{\left/ {\vphantom {{\left( {{\text{I}} - {\text{A}}} \right)} 2}} \right. \kern-0pt} 2}$$

7 $$\chi = {{\left( {{\text{I}} + {\text{A}}} \right)} \mathord{\left/ {\vphantom {{\left( {{\text{I}} + {\text{A}}} \right)} 2}} \right. \kern-0pt} 2}$$

This chemical hardness signifies resistance to deformation or polarization (of the electron cloud) surrounding atoms, ions, or molecules during a chemical reaction. Furthermore, hard molecules exhibit a more significant energy gap^31^. In contrast, soft molecules display the opposite characteristic and can be expressed as follows:

8 $$\sigma \left( {softness} \right) = \frac{1}{\eta }$$

9 $$\omega \left( {\text{electrophilicity index }} \right) = \frac{{\chi^{2} }}{2\eta }$$

The fraction of electrons (transferred to the metal surface) from the inhibitor molecule, (∆N) electrons^32^, can be determined using the formula as follows:

10 $$\Delta E_{{\text{back - donation}}} = \frac{ - \eta }{4}$$

11 $$\Delta N = \frac{{\left( {\chi_{Fe} - \chi_{inh} } \right)}}{{2\left( {\eta_{Fe} + \eta_{inh} } \right)}}$$

where “x_Fe_ and x_inh_ denote the absolute electronegativity of iron and inhibitor molecules, respectively. In addition, η_Fe_ and η_inh_ denote the absolute hardness of iron and inhibitor molecules, respectively. The theoretically derived values^33–35^ of η_Fe_ = 0 and χ_Fe_ = 7.0 eV”.

#### Monte-Carlo simulations

The optimal investigated compounds' configuration on the Fe (110) surface was examined via Monte Carlo simulations. “According to the existing literature, the Fe (110) crystal surface demonstrated the highest surface stability utilized for simulation^36^. To examine the adsorption of protonated and uncharged inhibitor molecules, water molecules (100) were inserted to trigger solvent impact in the solution (as a solvent layer) during the corrosion process. First, the quoting module was utilized to perform geometrical optimization on inhibitor and water molecules. The determination of adsorption sites characterized by minimized energy entails performing a Monte Carlo exploration of the configuration space for the substrate-adsorbate system while progressively reducing the temperature”. The energy of substrate-absorbate configurations, adsorption energy, and total energy were assessed through Monte Carlo simulations.

## Results and discussions

### Electrochemistry tests

#### Electrochemical impedance spectroscopy (EIS) tests

**Figure 1:**
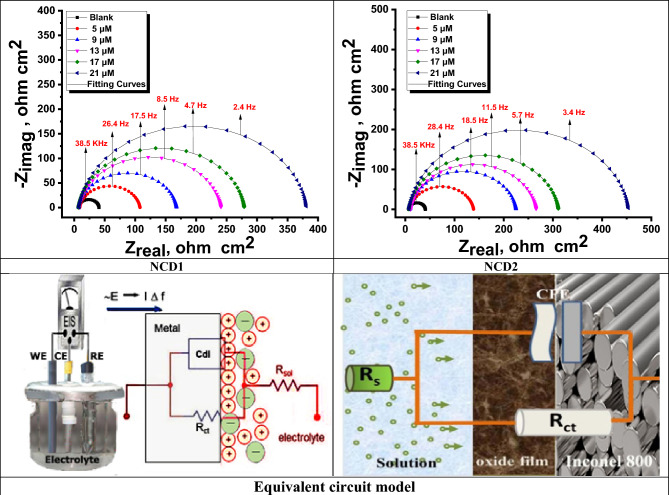
Nyquist curves for Inconel 800 in corrosive environment attendance and without altered doses of NCDs inhibitors at 25 °C and the equivalent circuit model used to fit the EIS data.

**Figure 2:**
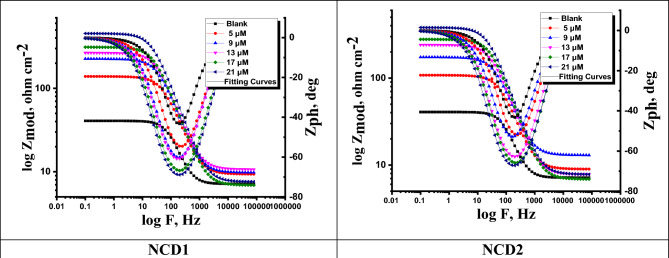
Bode diagrams for Inconel 800 in a corrosive environment without and with altered doses of NCDs at 25 °C.

The corrosion rate of Inconel 800 in a corrosive medium of 2.0 M HCl was examined utilizing EIS at a temperature of 25 oC. The investigation included various synthesized inhibitors' concentrations **NCD (1&2)** as well as without inhibitors. The Nyquist plot yields semi-circles as supposed in the theory of EIS (see Fig. 1). The EIS parameters were analyzed by fitting the equivalent circuit model shown in Fig. 1 which fits well with the experimental results. In Fig. 2 Bode phase diagrams, a comprehensive examination of the data reveals that each impedance plot displays a significant capacitive loop characterized by a capacitive time constant. The equivalent circuit compromises charge transfer resistance (R_ct_), constant phase element (CPE), and electrolyte resistance (R_s_). To eliminate the capacitance non-ideal response, CPE is utilized (as a condenser) in the electrochemical procedure^37^. The examined NCDs phase angle plots and bode are demonstrated in Fig. 2. The CPE outcome is determined based on the formula as follows:

12 $$Z_{CPE} = Y_{o}^{ - 1} \left[ {j\omega } \right]^{ - n}$$

With 0 ≤ n ≤ 1, j is an imaginary value and ω = 2πf while Y_o_ and f_max_, n are the CPE's magnitude, the frequency at which its maximum value is reached and the measure that reflects a deviation from the ideal behavior ranged from −1 to 1, respectively. Diffusion processes are characterized by the value of n = 0.5. The value of (n) represents the deviation from the ideal behavior and is found in the 0.911–0.980 interval revealing that the adsorbed inhibitor film is partially heterogeneous. The values of n, Y_o_, R_ct_ charge transfer resistance, C_dl_ double layer capacitance, and the η_EIS_ % were obtained and listed in Table 4. The decrease in Y_o_ is a consequence of a decrease in the local dielectric constant and/or an increase in the thickness of the double layer. The corresponding R_ct_ was also used to calculate η_EIS_% and CPE which is used to describe the double layer^38^.

13 $$C_{dl} = \left[ {Y_{o} R_{ct}^{1 - n} } \right]^{{{\raise0.7ex\hbox{$1$} \!\mathord{\left/ {\vphantom {1 n}}\right.\kern-0pt} \!\lower0.7ex\hbox{$n$}}}}$$

where (n) is a CPE exponent, Y_0_ is the CPE constant, and R_ct_ is the charge transfer resistance. The values of R_s_, C_dl,_ Y_0,_ R_ct,_ and exponent (n) are depicted in Table 3. According to the Nyquist plots obtained in the real system, the double layer on the metal/solution interface does not act as an aerial capacitor. The charge distribution is controlled by ions on the solution side and by electrons on the metal side. As ions are much larger than the electrons, the equivalent ions to the charge on the metal will occupy quite a large volume on the solution side of the double layer. Therefore, CPE is used in place of double layer capacitance, Cdl to represent the non-ideal capacitive behavior of the double layer. “The Nyquist and Bode plots exhibit alterations when subjected to diverse concentrations of the same inhibitor as well as distinct inhibitors. The diameter of the semicircle in the Nyquist plot progressively increased as the NCDs concentration increased, suggesting that the charge-transfer process primarily regulates Inconel 800 corrosion. Because the shapes of semicircles are the same, it means the mechanism of corrosion does not change during the process^39^. The Nyquist plot shape and loop diameter exhibit greater magnitudes in the presence of inhibitors compared to their absence. According to the data presented in Table 3, raising the concentration of the inhibitor leads to elevated resistance of the charge transfer R_ct_ values. This results in a significant difference between the resistance of the charge transfer and R_s_”. It is evident from the small chi-squared values presented in Table 3 that the fitted results exhibit a high level of congruence with the experimental data. The chi-squared metric was employed as a means to gauge the accuracy and precision of the fitting outcomes. The inhibition efficiency for the examined NCDs (utilizing the EIS method) is determined based on the R_ct_ values (using the formula as follows^40^:

14 $$\eta_{EIS} \% = \left[ {\frac{{\left( {R_{ct(inh)} - R_{ct} } \right)}}{{R_{ct(inh)} }}} \right] \times 100$$

where “R_ct_ is the electron charge transfer resistance (without NCDs) and R_ct(inh)_ is the electron charge transfer resistance (while using NCDs). Nevertheless, from the data displayed in Table 3, the R_ct_ values are elevated when using the inhibitors, whereas C_dl_ values are downregulated. The decline in C_dl_ values is attributable to inhibitor adsorption on the Inconel 800 surface as a result of the increasing double-layer thickness loop. The double layer formed between the charged Inconel 800 and the inhibitor solution is considered as an electrical capacitor. The adsorption of the inhibitor molecules on the Inconel 800 surface decrease the electrical capacity because they displace the water molecules and the ions are originally adsorbed on the surface. As per the EIS values obtained, the elevated inhibitor efficiency is reached at the maximum dose, and the most alleviated corrosion rate is achieved at the highest inhibitor dose”. Additionally, the protection order of the examined inhibitors are: **NCD2 > NCD1**.

**Table 3:** EIS outcome data for corrosion of Inconel 800 in the corrosive environment in the lack and presence of different doses of NCDs inhibitors at 25 °C.

Conc. (M)		Y_o_ (µ Ω^−1^ s^n^ cm^−2^) × 10^–6^	n	R_ct_, Ω cm^2^	C_dl_, μF cm^−2^	θ	η_EIS_ %	Goodness of fit (χ^2^)
2 M HCl		67.7	0.980	33	60	–	–	13.21 × 10^–3^
NCD1	5 × 10^–6^	43.8	0.941	99	31	0.667	66.7	14.57 × 10^–3^
	9 × 10^–6^	39.9	0.923	160	26	0.794	79.4	16.87 × 10^–3^
	13 × 10^–6^	34.4	0.917	233	22	0.858	85.8	12.35 × 10^–3^
	17 × 10^–6^	29.6	0.915	272	19	0.879	87.9	11.35 × 10^–3^
	21 × 10^–6^	27.1	0.911	373	17	0.912	91.2	13.87 × 10^–3^
NCD2	5 × 10^–6^	37.7	0.931	129	24	0.744	74.4	15.45 × 10^–3^
	9 × 10^–6^	32.5	0.922	215	21	0.847	84.7	12.74 × 10^–3^
	13 × 10^–6^	30.5	0.919	255	20	0.871	87.1	13.08 × 10^–3^
	17 × 10^–6^	27.6	0.915	304	17	0.891	89.1	14.11 × 10^–3^
	21 × 10^–6^	23.7	0.912	446	15	0.926	92.6	15.49 × 10^–3^

#### Measurements of potentiodynamic polarization (PDP)

**Figure 3:**
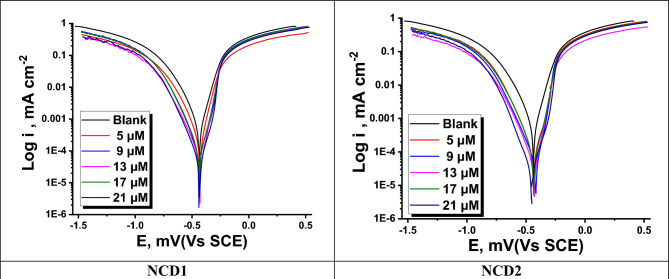
PDP bends for the corrosion of Inconel 800 in a corrosive environment in lack and attendance of altered concentrations of NCDs at 25 °C.

The correlation between the electrode potential and electric current during the polarization process can be elucidated by employing PDP investigations. The polarization tests may be employed to assess the progress of anodic and cathodic reactions, as well as inhibitors' impact on these reactions. Figure 3 plots the anodic and cathodic curves (from polarization) for the examined NCDs compounds at 25 °C. “The efficiency of the inhibition (η_PDP_ %), corrosion potential (E_corr_), anodic Tafel slope (β_a_), corrosion current density (I_corr_), and cathodic Tafel slope (β_c_)” are displayed in Table 4. η_PDP_ % was assessed^41^ as follows:

15 $$\eta_{PDP} \% = \left[ {1 - \frac{{i_{corr} }}{{i_{corr}^{^\circ } }}} \right] \times 100$$

**Table 4:** “Electrochemical results for Inconel 800 dissolution in the corrosive environment containing different doses of NCDs (1, 2) gotten from PDP measurements at 25 °C”.

Conc. (M)		i_corr_ (mA cm^−2^)	−E_corr_ (mV vs. SCE)	β_a_ (mV dec^−1^)	−β_c_ (mV dec^−1^)	C.R (mm y^−1^)	θ	η_PDP_ %
Blank		635	436	91	140	291	–	–
NCD1	5 × 10^–6^	167	430	72	117	76	0.737	73.7
	9 × 10^–6^	96	439	90	124	43	0.849	84.9
	13 × 10^–6^	60	429	88	126	27	0.906	90.6
	17 × 10^–6^	55	436	98	128	24	0.913	91.3
	21 × 10^–6^	52	433	94	123	21	0.918	91.8
NCD2	5 × 10^–6^	103	433	105	121	47	0.838	83.8
	9 × 10^–6^	53	417	88	127	24	0.917	91.7
	13 × 10^–6^	44	431	89	124	20	0.931	93.1
	17 × 10^–6^	41	435	93	123	19	0.935	93.5
	21 × 10^–6^	27	452	95	118	12	0.957	95.7

Table 4 shows that elevating the inhibitor concentration leads to a decrease in the corrosion rate (C.R) and (I_corr_) values. “Additionally, η_PDP_ % was elevated. This phenomenon is ascribed to the creation of a protective layer by the inhibitor, effectively impeding the active sites on the metal surface. Consequently, the dissolution of the anode material, Inconel 800, is restrained, and the evolution of hydrogen at the cathode is impeded. The E_corr_ values exhibit a marginal shift in the presence of inhibitors. The observed shift in value, typically < 85 mV, serves as a valuable metric for classifying the inhibitors under investigation as a mixed type. Notably, the change in E_corr_ values in the anodic and cathodic directions did not exceed 19 mV for all concentrations. The rise in surface coverage (θ) was observed as a result of the decrease in (I_corr_) values. This can be attributed to the development of an anodic protective layer (on the metal surface) induced by the elevated inhibitor concentration”. The inhibitors were investigated and found to have an inhibition following the order: NCD1 < NCD2.

### The inhibition efficiency & chemical measurements

Chemical tests were utilized to examine the IE% of the synthesized organic NCDs (1, 2). Inconel 800 corrosion rate was identified utilizing the gravimetric (weight loss) method.

#### Influence of concentration and temperature

**Figure 4:**
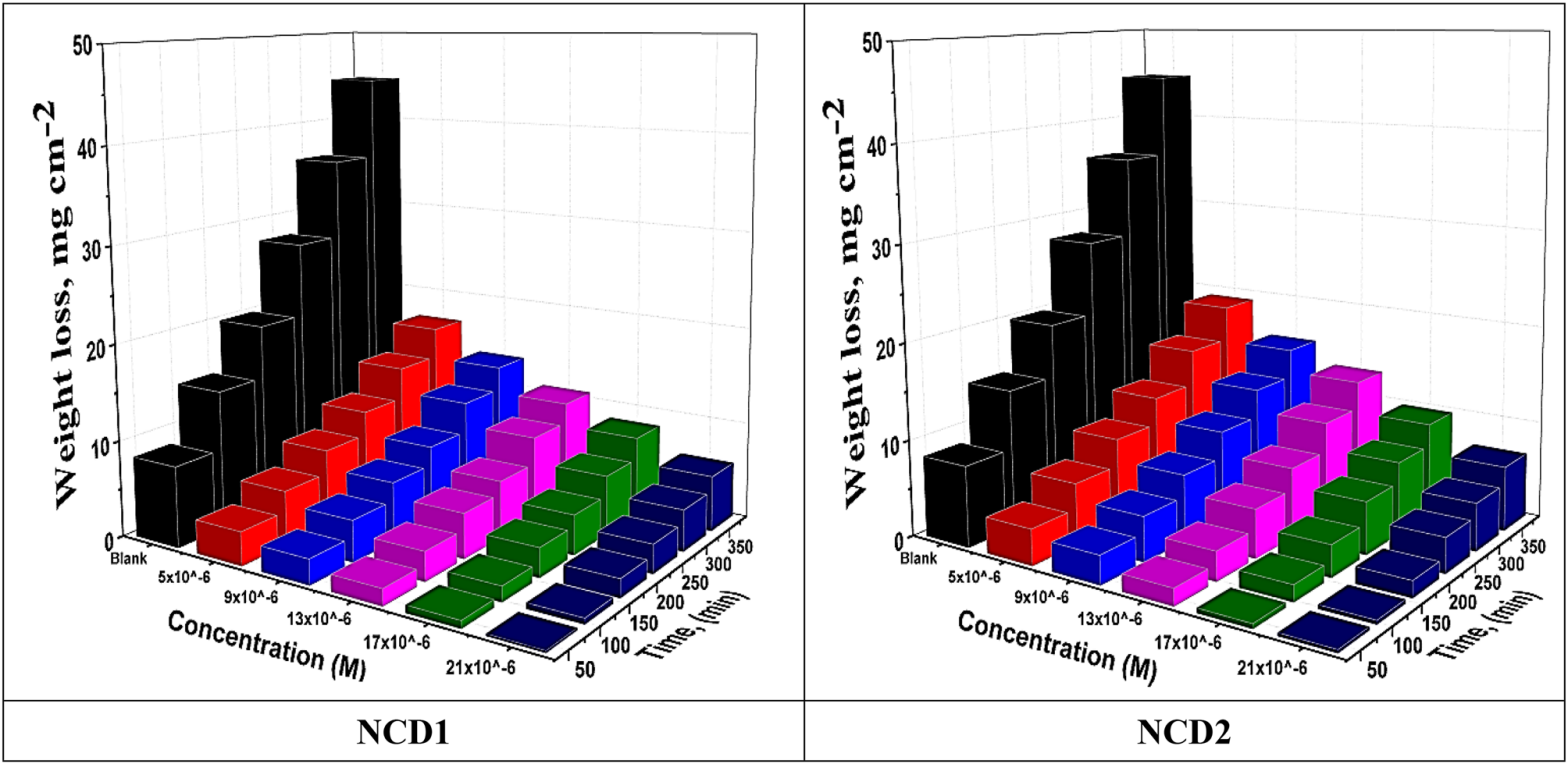
WL-time bends for dissolution of Inconel 800 in inhibited test solutions at altered doses of **NCDs** at 25°C.

For determining inhibitor concentration impact on the corrosion behavior, varying inhibitor concentrations (5–21 × 10^−6^ mol. L^−1^ range) were selected. Inconel 800 was dipped in a 2 M HCl solution with numerous inhibitor doses (6 h) to estimate corrosion rate as well as inhibition efficiency. WL-time curves of NCDs (1, 2) are shown in Fig. 4**,** which displays that WL decrease by increasing time from 1, 2, 3, 4, 5, and 6 h. “Corrosion parameters (obtained based on the WL method) at varying temperatures are depicted in Table 5, illustrating the estimated corrosion rate (C.R) values (mg cm^−2^ min^−1^), the (θ), and (η_w_ %) for Inconel 800 metal dissolution. A substantial decline was detected in the Inconel 800 corrosion rate, as well as elevated inhibition efficiency due to the elevation in inhibitor concentration. These results can be ascribed to the increased coverage of the Inconel 800 surface area with a rising amount of inhibitor, leading to enhanced inhibitor adsorption on the metal surface and hindrance of corrosion cells^42,43^. Consequently, the optimal inhibition efficiency and the lowest corrosion rate were detected at maximal concentration. The examined components’ absorption onto the Inconel 800 surface is facilitated through donor–acceptor interactions among the lone pairs of electrons on the N atoms and π-orbitals in aromatic rings, with the vacant d-orbitals of iron atoms. Additionally, the process involves displacing adsorbed H_2_O molecules from the metal surface. This displacement results in the blocking of active sites on the surface, leading to a reduction in the corrosion rate^44^. According to Table 5, the corrosion rate and inhibition efficiency reach the maximum with an inhibitor dose of 21 × 10^−6^ mol L^−1^ in the solution indicates the optimum added inhibitor dose. Consequently, any treatment aiming to surpass the maximum inhibitor dose is deemed unnecessary”. The maximal inhibition efficiency is recorded at 93.2% and 91.6% for NCD2 and NCD1, respectively. The disparity in compounds' efficiency is attributed to the number of existing π-orbitals and heteroatoms in the compounds^45^.

**Table 5:** Corrosion criteria (C.R and η_w_ %) from WL of Inconel 800 dissolution in the corrosive environment in the presence and non-presence of **NCDs** at the studied temperatures after 240 min.

Temp (°C)	[inh.] × 10^–6^ M	NCD1		NCD2	
		C.R (mg/cm^2^/min)	η_w_ (%)	C.R (mg/cm^2^/min)	η_w_ (%)
25	Blank	0.191	–	0.191	–
	5	0.061	68.1	0.051	73.3
	9	0.052	72.8	0.032	83.2
	13	0.042	78.0	0.025	86.9
	17	0.034	82.2	0.021	89.0
	21	0.016	91.6	0.013	93.2
35	Blank	0.245	–	0.245	–
	5	0.082	66.5	0.073	70.2
	9	0.074	69.8	0.061	75.1
	13	0.063	74.3	0.055	77.6
	17	0.054	78.0	0.046	81.2
	21	0.039	84.1	0.031	87.3
45	Blank	0.283	–	0.283	–
	5	0.101	64.3	0.091	67.8
	9	0.092	67.5	0.075	73.5
	13	0.071	74.9	0.063	77.7
	17	0.063	77.7	0.052	81.6
	21	0.052	81.6	0.043	84.8
55	Blank	0.355	–	0.355	–
	5	0.132	62.8	0.121	65.9
	9	0.121	65.9	0.114	67.9
	13	0.118	66.8	0.105	70.4
	17	0.082	76.9	0.075	78.9
	21	0.074	79.2	0.064	82.0
65	Blank	0.496		0.496	
	5	0.234	52.8	0.185	62.7
	9	0.201	59.5	0.165	66.7
	13	0.186	62.5	0.134	73.0
	17	0.154	69.0	0.116	76.6
	21	0.122	75.4	0.097	80.4

#### Temperature impact

**Figure 5:**
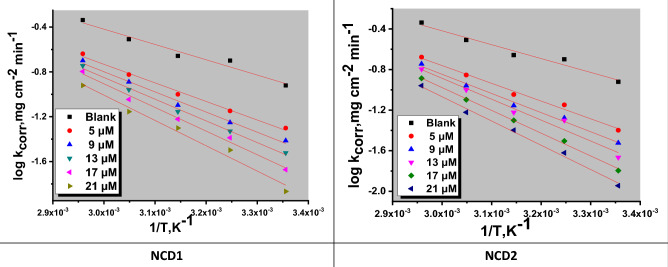
Arrhenius bends for the dissolution of Inconel 800 in a corrosive solution in non-presence and with several doses of NCDs (1&2).

**Figure 6:**
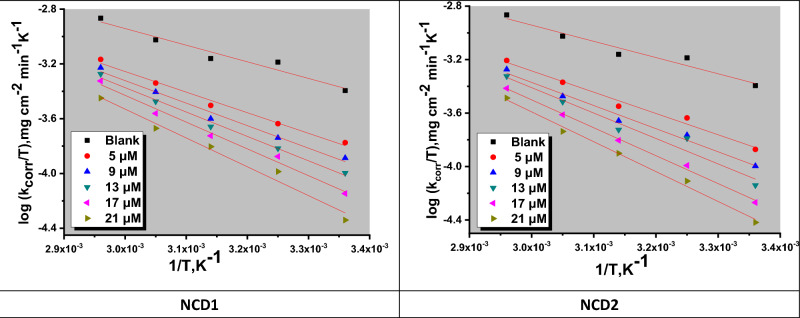
Transition curves for the dissolution of Inconel 800 in corrosive media in non-presence and presence of several doses of NCDs (1&2).

Temperature influence on Inconel 800 corrosion behavior (with and without NCDs) using varying doses was examined at temperatures 338, 328, 318, 308, and 298 K (utilizing WL measurements). “According to Table 5, it is evident that the corrosion (in the absence of inhibitors) significantly intensifies as the temperature rises from 298 to 338 K, whereas the corrosion rate only marginally increases when inhibitors are present. Moreover, corrosion inhibition effectiveness diminishes as the temperature rises, demonstrating that the corrosion protection ability is better preserved at lower temperatures. This phenomenon can be attributed to the rise in temperature, which reduces the interactions among the inhibitor molecules and the Inconel 800 surface. Consequently, the desorption of these molecules from the surface of Inconel 800 is enhanced at high temperatures. This temperature influence suggests that the adsorption is of a physical adsorption type”^46^. The impact of temperature on the corrosion rate can be elucidated by employing the Arrhenius balance^47^.

16 $$\log k_{corr} = \left( {\frac{{ - E_{a}^{ * } }}{2.303RT}} \right) + \log A$$

while R signifies the universal gas constant E_a_^*^ is the apparent activation energy, “In Fig. 5, a linear graphical representation is presented, illustrating the correlation between the natural logarithm of the corrosion rate (log k_corr_) and the reciprocal of the absolute temperature (1/T) for Inconel 800 immersed in a 2 M HCl solution. Through an examination of the gradient values at various temperatures, it becomes feasible to ascertain the Arrhenius activation energy (E_a_^*^). The application of the Arrhenius-type model facilitated the computation of kinetic parameters associated with the corrosion of Inconel 800 In addition, the elevation of Ea signifies the augmentation of the energy barrier, thereby promoting the interaction between the inhibitor molecule and iron atoms (on the metal surface)^48^. Additional thermodynamic parameters, including entropy (ΔS*) and enthalpy (ΔH*)”, were calculated utilizing the transition state equation^49^:

17 $$\log k_{corr} = \log \left( \frac{R}{Nh} \right) + \frac{{\Delta S^{ * } }}{2.303R} + \frac{{\Delta H^{ * } }}{2.303RT}$$

while R signifies the universal gas constant, “N denotes Avogadro's number, and h stands for the Planck constant. The graphical representation of the relationship between the logarithm of the rate constant (log k_corr_) divided by the absolute temperature (T) and the reciprocal of temperature (1/T) produces linear plots characterized by slopes equivalent to (ΔH^*^ / 2.303R) and intercepts denoted as [log (R/Nh + ΔS^*^/2.303R)], as illustrated in Fig. 6, and are presented in Table 6. Furthermore, the positive values of ΔH^*^ confirm that the formation of the activated complex is an endothermic process^50^. In contrast, ΔS* negative sign suggests that the stage of activated complex formation in the rate-determining step inclined more towards association than dissociation”^51^. Consequently, the decline in disordering facilitates the development of the activated complex^52^.

**Table 6:** Thermodynamic parameters for Inconel 800 dissolution in the corrosive environment at several doses of 1 NCDs (1&2) calculated from WL assessments.

Inhibitor	Conc. (× 10^–6^)	Activation parameters		
		E_a_* (kJ mol^−1^)	ΔH* (kJ mol^−1^)	−ΔS* (J mol^−1^ K^−1^)
Free acid (2 M HCl)		26.1	23.2	184
NCD1	5	31.7	28.7	173
	9	34.4	31.4	166
	13	37.1	34.1	159
	17	40.4	37.3	150
	21	43.2	40.2	144
NCD2	5	33.4	30.4	168
	9	36.1	33.1	162
	13	39.4	36.4	153
	17	42.9	39.9	144
	21	45.7	42.7	137

#### Adsorption isotherm study

The interaction between NCDs inhibitor and Inconel 800 surface is governed by numerous adsorption isotherms such as Langmuir, Temkin, Henry, and Friendlish which are the most frequently used^53^. The correlation coefficient (R^2^) values are close to unity, and thus, the examined inhibitors conform to the Langmuir adsorption isotherm. It is worth noting that among these models, the Langmuir isotherm exhibited the most favorable fitting characteristics, yielding the best linear plots, all these adsorption isotherms are described by the following equation^54^:

18 $${\text{Langmuir}} \quad \frac{{C_{inh} }}{\theta } = \frac{1}{{K_{ads} }} + C_{inh}$$

19 $${\text{Temkin}}\quad \alpha \theta = \ln K_{ads} C$$

20 $${\text{Henry}}\quad \theta = K_{ads} C$$

21 $${\text{Friendlish}}\quad \log \theta = \log K_{ads} + n\log C$$

**Figure 7:**
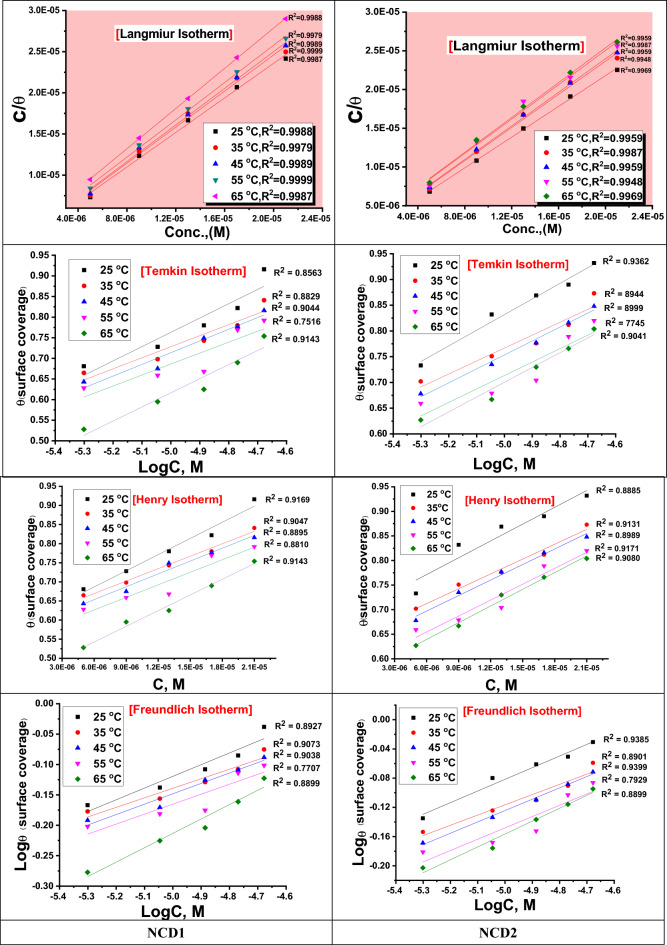
Different adsorption isotherm bends for the Inconel 800 dissolution in a corrosive environment in the presence of NCDs inhibitor at altered temperatures.

“The weight loss method was employed to ascertain the adsorption constant (K_ads_), inhibitor concentration (C), α is a factor describing the molecular interactions in the adsorption layer & the heterogeneity of the surface, and surface coverage (θ) for various concentrations in a scholarly investigation. In all cases, the degree of surface coverage obtained from WL measurements is plotted as a function of the concentration of prepared NCDs. By calculating the correlation coefficients (R^2^) of the straight lines, we can able to choose the best adsorption isotherm model that fits the experimental data. The most appropriate isotherm, which exhibited the highest correlation (R^2^) with the experimental data, was selected. The observed strong correlation (R^2^ = 0.99) strongly suggests that the adsorption of NCDs onto the Inconel 800 surface closely adhered to this selected isotherm. The graphical representations in Fig. 7 displayed linear plots of different adsorption isotherms, implying that the NCDs adhered to the metal surface following the Langmuir adsorption isotherm (the strong correlation R^2^ is almost nearly 1). According to this particular isotherm, there exist no intermolecular interactions among the adsorbed species, each occupying a discrete site”. Moreover, in accordance with the subsequent relationship^55^, the equilibrium constant (K_ads_) governing the adsorption process is intricately linked to the free energy of adsorption (∆G^o^_ads_).

22 $$K_{ads} = \left( {\frac{1}{55.5}} \right)\exp \left( {\frac{{ - \Delta G_{ads}^{^\circ } }}{RT}} \right)$$

The term "mol L^−1^ (M)" denotes the molar concentration of water within the solution, specifically at a value of 55.5 in the present context. “As per the calculated data presented in Table 7, it is observed that NCDs exhibit a notable and substantial chemical adsorption affinity on Inconel 800 when immersed in a solution containing 2 M HCl. This conclusion is derived from the determined Gibbs free energy of adsorption (∆G^o^_ads_) values. The ΔG°_ads_ values can indicate adsorption process type, ΔG^°^_ads_ up to −20 kJ mol^−1^ correlation with physisorption, involving the electrostatic interface among charged inhibitor's charged metal surface and molecules^56^. In contrast, magnitudes more negative than −40 kJ mol^−1^ are linked to chemisorption, suggesting the coordination interface between the metal surface and inhibitor molecules^57,58^. In this work, the estimated ΔG^°^_ads_ values for all inhibitors exhibited large negative values around − 40 kJ mol^−1^ and elevated with the temperature. This finding indicates that inhibitors' spontaneous chemisorption on the Inconel 800 surface occurs and strengthens by down-regulating the temperature”. The heat of adsorption (ΔH^°^_ads_) can be determined based on the Van't Hoff equation^59^.

23 $$\Delta G_{ads}^{^\circ } = \Delta H_{ads}^{^\circ } - T\Delta S_{ads}^{^\circ }$$

**Table 7:** The calculated parameters from the Langmuir adsorption isotherms of NCDs at different temperatures after 240 min.

Inhibitor	Temp (°C)	R^2^	K _ads_ (M^−1^)	−ΔG°_ads_ (kJ mol^−1^)	−ΔH°_ads_ (kJ mol^−1^)	ΔS°_ads_ (J mol^−1^ k^−1^)
NCD1	25	0.9988	382	24.69	5.9	63
	35	0.9979	370	25.43		
	45	0.9989	361	26.19		
	55	0.9999	319	26.68		
	65	0.9987	289	27.22		
NCD2	25	0.9959	514	25.42	8.7	56
	35	0.9987	443	25.89		
	45	0.9959	435	26.69		
	55	0.9948	346	26.90		
	65	0.9969	345	27.71		

**Figure 8:**
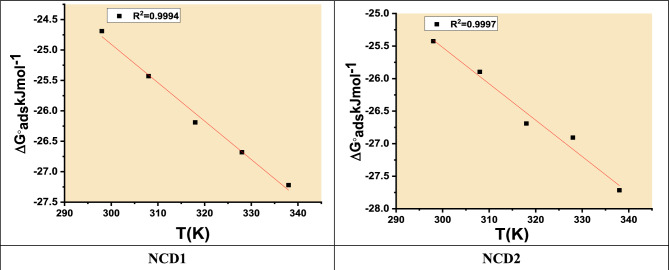
ΔG°_ads_ versus T for NCDs inhibitor.

ΔG°_ads_ graphs against T for NCDs are displayed in Fig. 8. “From the slope (− ∆S°_ads_) of the achieved lines and intercept equal to (ΔH°_ads_ as illustrated in Table 7. From ΔH°_ads_ obtained values, the corrosion inhibition mechanism can be elucidated. Conversely, the positive ΔH°_ads_ values refer to the endothermic adsorption mechanism. Typically, the chemisorption mechanism is linked to the endothermic process. Nevertheless, the exothermic process might be associated with chemisorption and physisorption (or both together)^60^. Physisorption is distinguished from chemisorption in an exothermic process by considering that the exothermic value of a physisorption process is lower than −20 kJ mol^−1^ while the chemisorption heat of the process should be lower than −41 kJ mol^−1^. A negative value of ΔH°_ads_ indicated that the inhibitor's adsorption process is exothermic and the adsorption process may be physical or chemical. In our study, the ∆G°_ads_ value is 25.4 kJ mol^−1^ and ΔH°_ads_ is −5.9 kJ mol^−1^, suggesting that the adsorption is of mixed type. Due to NCDs adsorption on the surface of Inconel 800, the standard adsorption entropy's positive value indicated that the adsorption was associated with an increase in system ordering^61^. Table 7 presents the data from the Langmuir adsorption isotherm. The spontaneity of the adsorption process and the stability of the adsorbed layer on the metal surface are both ensured by the negative ∆G°_ads_ values”. In aqueous media, the range of ∆G°_ads_ for mixed adsorption processes for organic inhibitors is typically between −24.69 and −27.71 kJ mol^−1^^62^_**.**_

### Morphological analysis for the surface

#### SEM with EDX analysis

**Figure 9:**
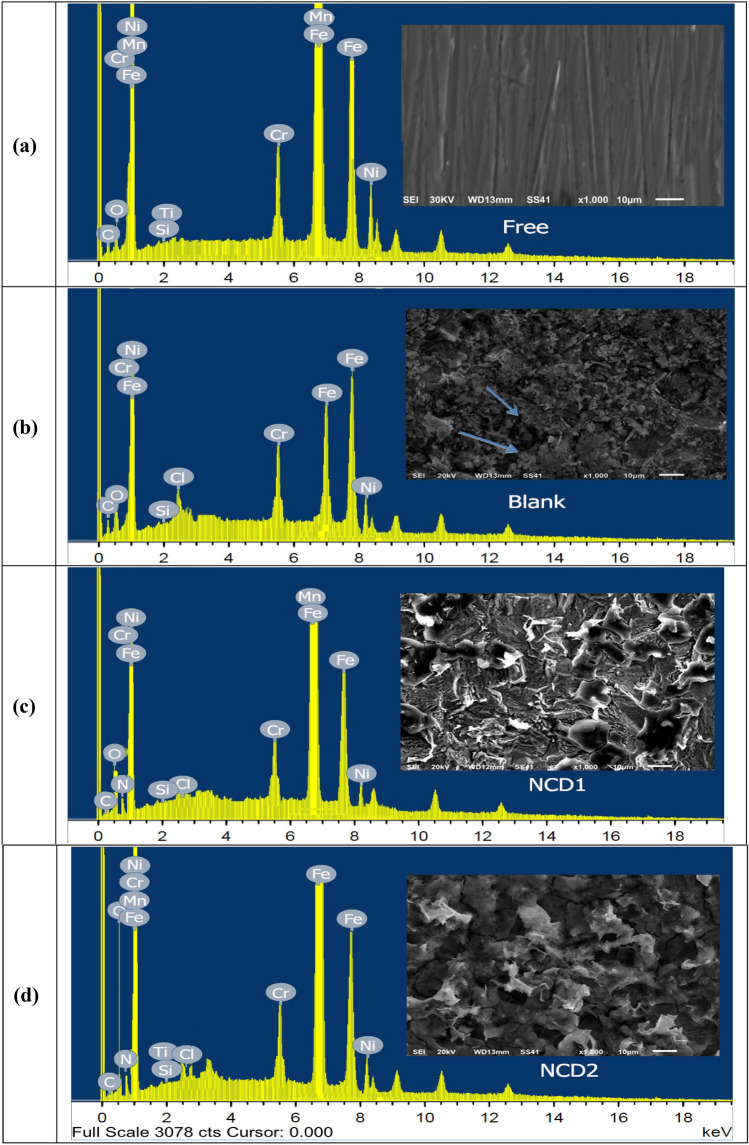
SEM profiles and EDX for: (**a**) Inconel 800 free, (**b**) following a 24-h immersion in a 2.0 M hydrochloric acid solution without inhibitors, (**c**) with NCD1, and (**d**) presence of NCD2 inhibitors.

Inconel 800 specimen surface morphology was examined utilizing scanning electron micrographs before and following dipping in HCl 2.0 M for 24 h (with and without) inhibitors at ambient temperature. Figure 9a–d depicts the SEM and EDX images. For SEM (Fig. 9a) Inconel 800 before, (Fig. 9b) following placing in HCl, Fig. 9**c** following placing in inhibitor (**NCD1**), and Fig. 9d following placing in inhibitor (**NCD2**), respectively. Upon observation, it is evident that the surface of Inconel 800 before being exposed to acid is significantly smoother compared to the surface immersed in HCl (without inhibitors). The latter surface shows extensive damage, with numerous dark spots covering it, resulting from the aggressive attack of the acid solution (on the unprotected surface)^63^. In contrast, Inconel 800 samples obtained from the solutions containing inhibitors exhibit a relatively smooth surface despite the presence of a corrosive substance. This is because the solution's inhibitor lowers the rate of corrosion by creating a layer of protection between the corrosive material and the surface of Inconel 800. As a result, the roughness decreases, and the surface morphology improves^64^, as demonstrated in Fig. 9c, d. The observed surface scratches are primarily caused by polishing abrasions resulting from mechanical wear. The smoothness of the surface improves in correlation with the effectiveness of inhibitors. The surface treated with inhibitor (**NCD2**) is notably smoother. Analysis of energy dispersive X-rays (EDX) revealed information regarding the atom of NCDs. The spectrum is shown in Fig. 9a–d. In the case of Inconel 800 immersed in 2.0 M HCl only, the Fe and Cl can only detect Fig. 9b. The data indicates that the percentage of atoms measured experimentally appears to be generally comparable to the theoretical or expected values. The presence of N, O, and C is readily established by the EDX spectrum of the NCDs, which displays specific signals corresponding to carbon, oxygen, and nitrogen Fig. 9c, d in which both the SEM image and EDX data designated that NCDs have formation of protective layer adsorbed on in Inconel 800 surface.

#### FT-IR spectra

**Figure 10:**
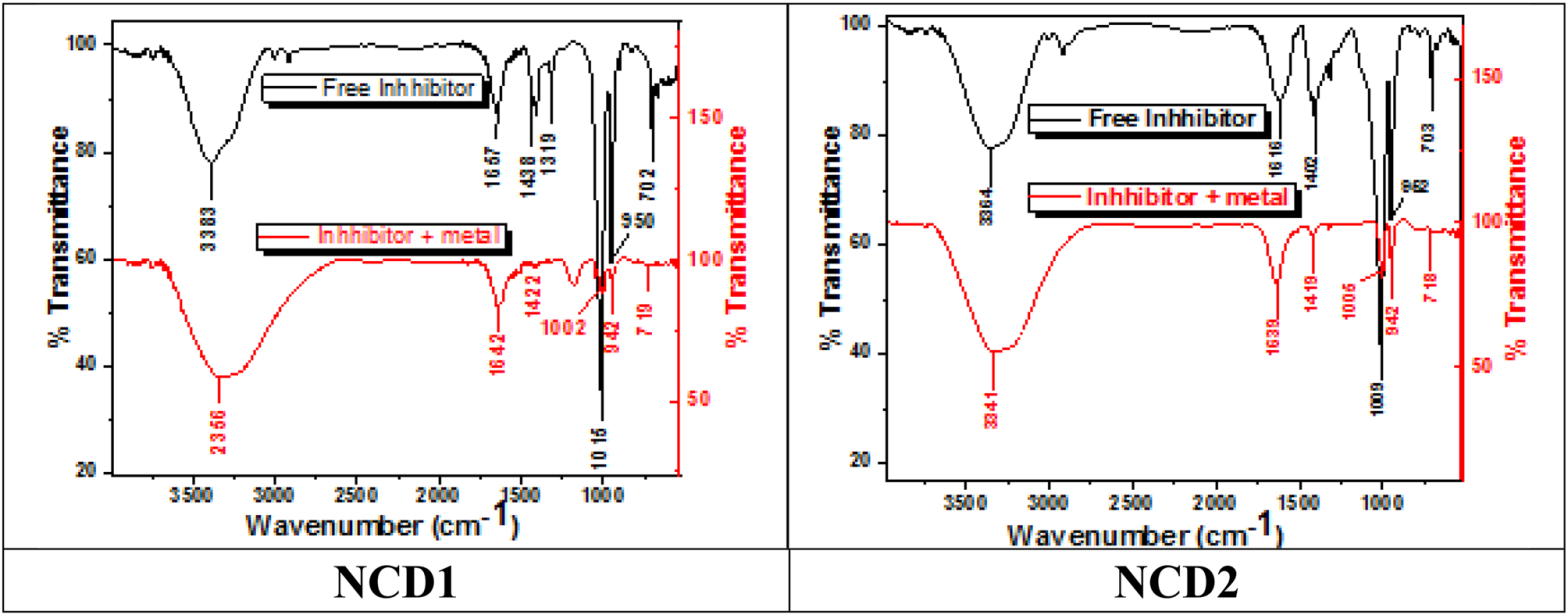
FT-IR spectra of pure inhibitors and the Inconel 800 surface were compared after a 24-h immersion in a 2.0 M HCl solution with 21 $\times$ 10^–6^ M of NCD (1, 2) at 298 K.

It is used to identify the functional groups of both pure NCDs and adsorbed on the surface of Inconel 800 as well as indicate the strong coordination bonds that formed between these groups and Inconel 800 metal. Figure 10 shows IR spectra of both refined grinded NCDs inhibitor and adsorptive inhibitors on Inconel 800 in the presence of 2 M HCl. Figure 10 (NCD1), the –OH stretching has moved from 3383 to 3356 cm^−1^. The C=C stretch has moved from 1657 to 1642 cm^−1^. The N=N stretching has moved from 1438 to 1422 cm^−1^. The (C–O) stretch has moved from 1015 to 1002 cm^−1^. Figure 10 (NCD2), the peaks corresponding to O–H stretch at 3364 cm^−1^ shift to 3341 cm^−1^, and the peaks attributed to C=C stretch at 1616 cm^−1^ shift to 1639 cm^−1^, and the peaks of N=N stretch at 1402 cm^−1^ showed shift at 1419 cm^−1^, and the peaks shifted from 1009 cm^−1^ to1005 cm^−1^ can be attributed to (C–O). By making well comparison between these spectra, it is found that they have matching characters which is considered conclusive evidence of NCD (1&2) adsorption on Inconel 800 surface, the different function groups and shifts which illustrate the interaction between organic inhibitors and Inconel 800 surface forming protective coating thin layer on Inconel 800 surface which has been confirmed by the lower intensity of peaks^65,66^.

#### AFM tests

**Figure 11:**
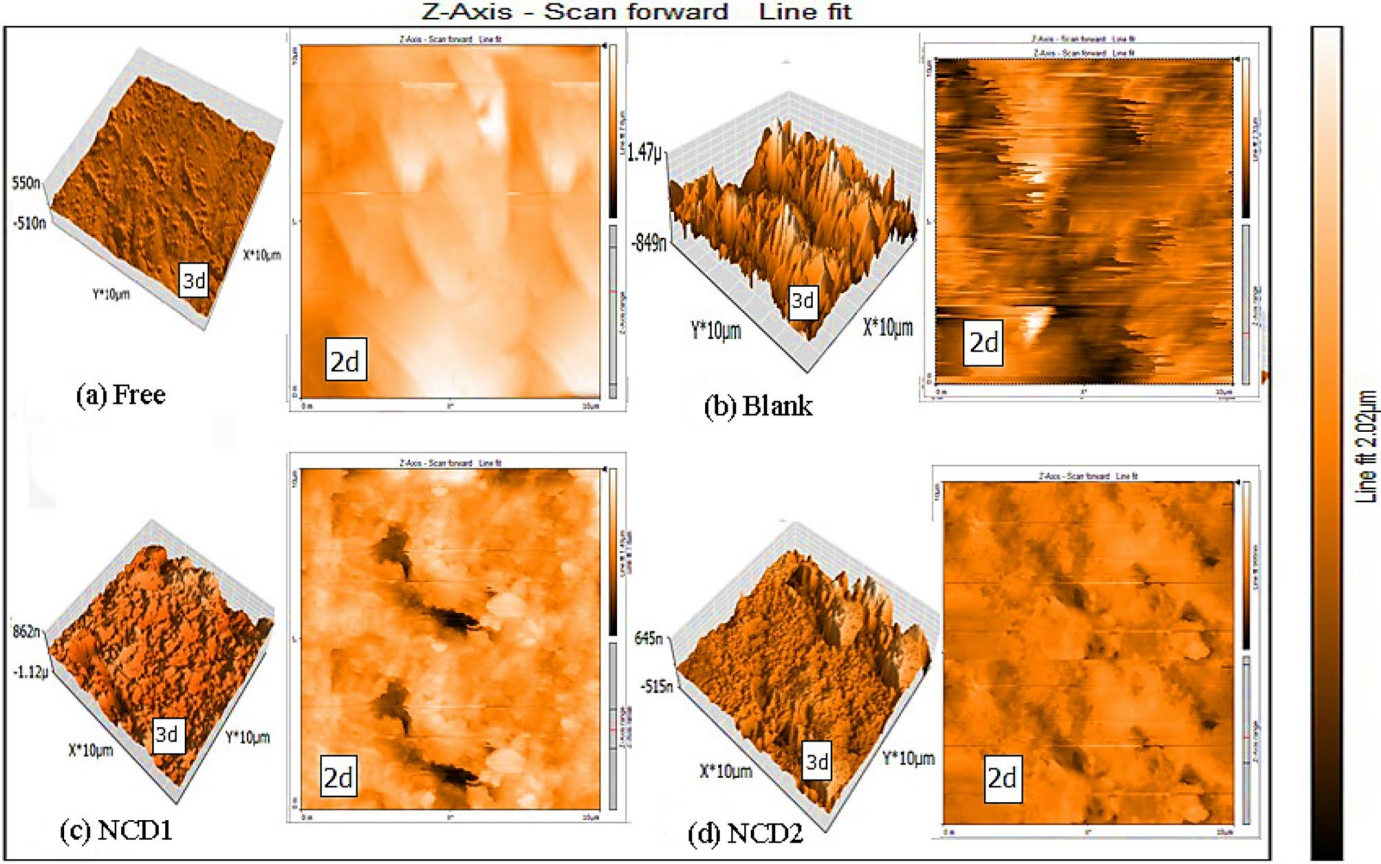
AFM (2D and 3D) image of the Inconel 800 (**a**) before dipping, (**b**) Inconel 800 dipping in solution test for 24 h without NCDs, (**c,d**) dipping in corrosive solution for 24 h in the existence NCD(1, 2).

AFM is becoming an accepted method for investigation of the roughness of metals, and alloys. The two and three-dimensional AFM images for Inconel 800 surface (free) for 24 h at 298 K (see Fig. 11a) show low surface roughness at 19.3 nm, after keeping Inconel 800 in (2 M HCl) Fig. 11b high surface roughness reached 618.13 nm, while in the attendance of (2 M HCl + 21 $\times$ 10^–6^ mol. L^−1^ NCD (**1, 2**) mixture decreased to 160.8 nm and 110.7 nm correspondingly (see Fig. 11c,d). The decrease in average surface roughness of Inconel 800 in existence of NCD (**1, 2**) are due to the adsorption of these NCD (**1, 2**) on Inconel 800 surface forming protective layer, as a result, the metal's surface smoothed out and the rate of corrosion dropped.

### Computational methods

#### Quantum chemical calculations

**Figure 12:**
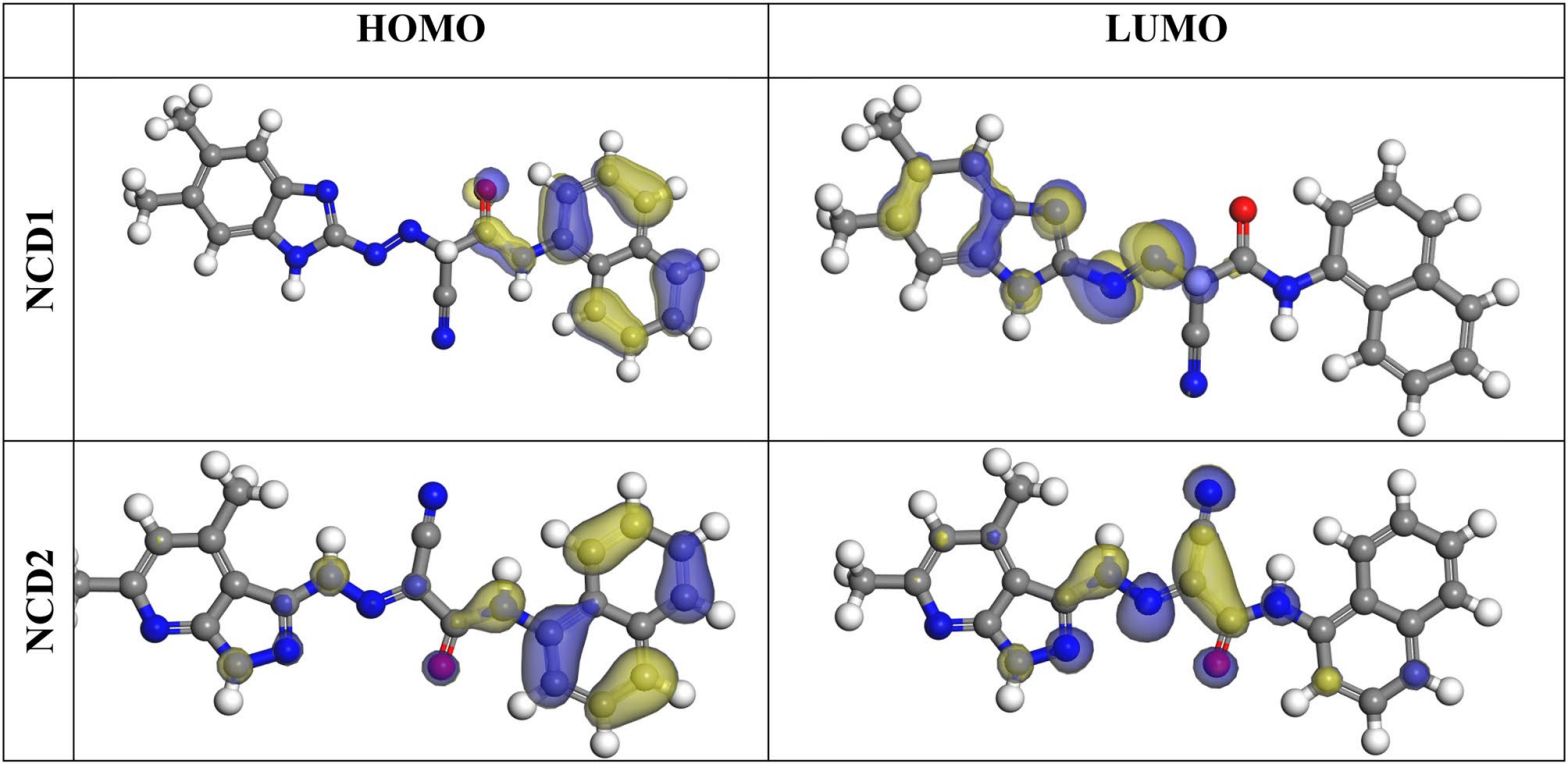
HOMO and LOMO calculated for the tested NCDs at DMol3.

The optimized structures of LOMO and HOMO are presented in Fig. 12, obtained utilizing DMol3 with the DFT method. “E_HOMO_ signifies the capability for electron donation, while E_LUMO_ denotes the capability for electron acceptance. A molecule is more able to donate electrons if its E_HOMO_ is higher and its negative energy value is smaller. Furthermore, the molecule is far more probable to receive electrons when the magnitude of E_LUMO_ is negative^67^. The adsorption capacity of inhibitor molecules on the metal surface elevates with a greater E_HOMO_ value and a decreased E_LUMO_ value, thereby enhancing corrosion inhibition efficiency^68^. The energy gap (ΔE), defined as the difference between HOMO energy and LUMO energy, is a crucial parameter. The literature generally recognizes a relationship between %IE and the energy gap (ΔE), where molecules with a smaller energy gap value exhibit enhanced inhibition efficiency. Table 8 lists the theoretical parameters that have been determined. Higher dipole moment values enhance adsorption between the inhibitor and the metal surface (Debye). As shown in Table 8, NCD2 has the most elevated ΔN value, indicating a higher ability to share electrons with the Inconel 800 surface than NCD1. Furthermore, NCD2 has a lower energy gap (ΔE) than NCD1, demonstrating a superior ability for adsorption on the Inconel 800 surface and elevated efficiency as a corrosion inhibitor. Computational measurements indicate that the inhibitors under investigation are adsorbed (on the surface of Inconel 800) by donating electrons or charges to the iron vacant orbitals. Furthermore, an inhibitor molecule's molecular surface area plays a critical role in determining its capacity to protect metal surfaces from corrosive environments. Because the inhibitor molecules cover a larger surface area on the Inconel 800, the inhibitory efficacy improves as the molecular structure gets larger. Therefore, as shown in Table 8, the NCD2 composite has the highest molecular surface area (457.874 Å^2^) and, as a result, greater inhibition proficiency for a-NCD2 than NCD1 (445.418 Å^2^)”. Additionally, the quantum parameters the following order of reactivity and inhibition efficiency: NCD2 > NCD1, as experimentally demonstrated.

**Table 8:** Quantum chemical data for the inhibitors under consideration.

Compound	NCD1	NCD2
E_HOMO_, eV	−5.082	−4.896
E_LUMO_, eV	−4.079	−4.138
ΔE, eV	1.003	0.758
I_P_, eV	5.082	4.896
E_A_, eV	4.079	4.138
η, eV	0.502	0.379
σ, eV	1.994	2.64
ω, eV	20.92	26.92
∆N	2.41	3.28
Δ*E*_back-donation_	−0.13	−0.09
Dipole moment (μ) Debye	7.24	8.21
Molecular surface area, Å^2^	445.418	457.874

#### Fukui function

**Figure 13:**
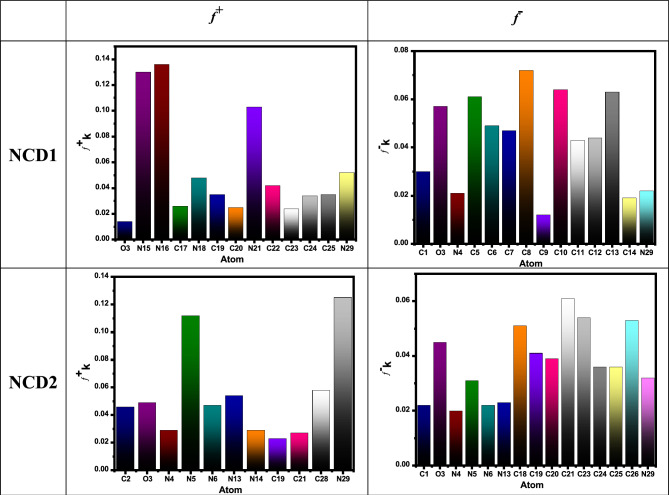
Fukui indices (${f}_{k}^{+}$ is a nucleophilic attack, ${f}_{k}^{-}$ is an electrophilic attack) calculated for the tested NCDs at DMol3.

It is an important technique for knowing how the adsorption process on the Inconel 800 surface can occur. Its main roles are an indication of both reaction sites which are electrophilic and nucleophilic sites, as well as, the determination of atoms that attack the metal as electrophile or nucleophile^69^. Electrons transfer from nucleophilic to electrophilic sites to illustrate reactive sites. The tendency of molecules to donate or accept electrons is demonstrated by knowing the number of nucleophilic sites. Figure 13 displays Fukui indices, which are calculated by the following equations^70^:

24 $$f_{k}^{ + } = [q_{k} (N + 1) - q_{k} (N)]$$

25 $$f_{k}^{ - } = [q_{k} (N) - q_{k} (N - 1)]$$

26 $$\Delta f_{k} = f_{k}^{ + } - f_{k}^{ - }$$

where, ${f}_{k}^{+}$ is a nucleophilic attack, ${f}_{k}^{-}$ is an electrophilic attack and $\Delta {f}_{k}$ is the dual descriptor. If $\Delta {f}_{k}>0$, the atoms are electrophile. In contrast, if $\Delta {f}_{k}<0$, the atoms are nucleophiles. If the value of ${f}_{k}^{+}$ of atom increase, this atom is considered as the desired site for nucleophilic attack. On the other hand, if ${f}_{k}^{-}$ of atom increase and become the greatest value, this atom is considered as the desired site for electrophile attack which is originally extended over Inconel 800 surface^71^.

The local reactivity of the organic compounds can be evaluated by reckoning the Fukui directories (${{\varvec{f}}}_{{\varvec{k}}}^{+}$ and ${{\varvec{f}}}_{{\varvec{k}}}^{-}$), the local electrophilicity (${\upomega }_{{\varvec{k}}}^{\boldsymbol{ }\pm }$), the local softness descriptor (${{\varvec{\upsigma}}}_{{\varvec{k}}}^{\boldsymbol{ }\pm }$), and the dual descriptors (Δ*f*_*k*_, Δ*σ*_*k*_, and Δ*ω*_*k*_) from the following equations^72–74^:

27 $$\sigma_{k}^{ \pm } = \sigma f_{k}^{ \pm }$$

28 $$\omega_{k}^{ \pm } = \omega f_{k}^{ \pm }$$

29 $$\Delta f_{k}^{ \pm } = f_{k}^{ + } - f_{k}^{ - }$$

30 $$\Delta \sigma_{k} = \sigma_{k}^{ + } - \sigma_{k}^{ - }$$

31 $$\Delta \omega_{k}^{ \pm } = \omega_{k}^{ + } - \omega_{k}^{ - }$$

**Figure 14:**
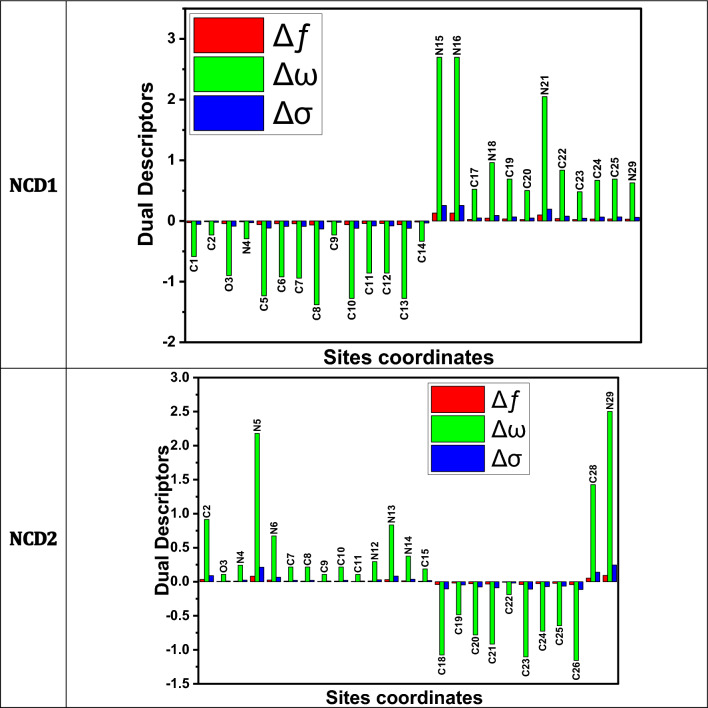
Graphical depiction of the dual descriptors (Δ*f*, Δ*σ*, and Δ*ω*) for the maximum active centers of the studied organic molecules using the DFT method.

For clarification, the most meaningful results are revealed in Table S1. “The evaluated Fukui directories (Table S1) detected for the inhibitor species are ascribed to the sites at which the organic molecules will be adsorbed onto the Fe interface. Furthermore, the local dual descriptors are more accurate and comprise more tools than the Fukui directories (${{\varvec{f}}}_{{\varvec{k}}}^{+}$ and ${{\varvec{f}}}_{{\varvec{k}}}^{-}$), the electrophilicity (${\upomega }_{{\varvec{k}}}^{\boldsymbol{ }\pm }$), and the local softness (${{\varvec{\upsigma}}}_{{\varvec{k}}}^{\boldsymbol{ }\pm }$),; the graphical demonstration of the dual local descriptors of the greatest illustrative active centers is displayed in Fig. 14. The attained outcomes show that the sites with the Δ*f k*, Δ*σk*, and Δ*ωk* < 0 have the propensity to relocate electrons to the steel surface. On the other hand, those sites with Δ*f k*, Δ*σk*, and Δ*ωk* > 0 have the ability to accept an electron from the steel. As can be seen in Fig. 14, the highest active centers for electron donation are at C1, C2, O3, N4, C12, C5-C14 for NCD1; and C18-C26 for NCD2. The active accepting centers are at N15, N16, C17, N18, C19, C20, N21, C22-C25, N29 for NCD1; and C21, O3, N4-N6, C7-C11, N12-N15, C15, C28, N29 for NCD2”.

#### Molecular electrostatic mapping potential (MEP)

**Figure 15:**
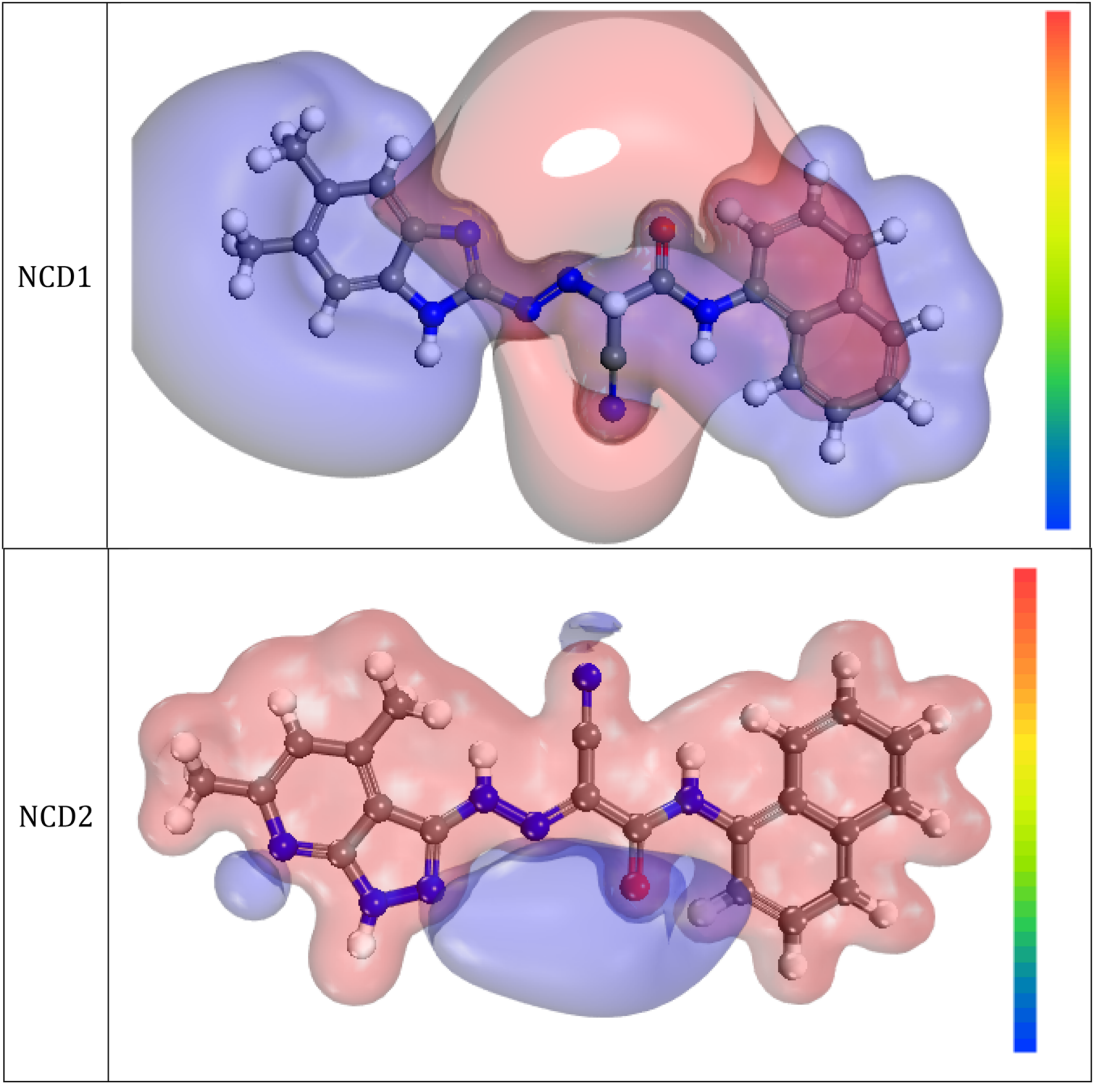
Graphical presentation of the MEP of the organic molecules using the DFT method.

Finally, molecular electrostatic mapping potential (MEP) could divulge the efficient sides of the organic molecules and is evaluated through the Dmol3 module. “It indicates that organic inhibitor has a transparent surface for both total electron density and MEP images show many sites and centers with negative and positive potential. The MEP map is a 3D image descriptor aimed at discriminating the net electrostatic influence originating on a compound by the complete charge sharing^75^. In MEP mapping revealed in Fig. 15, the red area depicts the great electron density extent; where the MEP is exceedingly negative (nucleophilic interaction). In contrast, the blue area designates the maximum positive zone (electrophilic attraction)^76^. A visual investigation of Fig. 15 supports that the extreme negative portions are mainly above nitrogen and oxygen atoms; however, there is a lower electron density over the aromatic system (benzene rings)”. These centers with greater electron density (i.e., red zone) in additive molecules may be the best for adsorption on the steel surface, creating durable adsorbed protecting films.

#### Monte Carlo (MC) simulation

**Figure 16:**
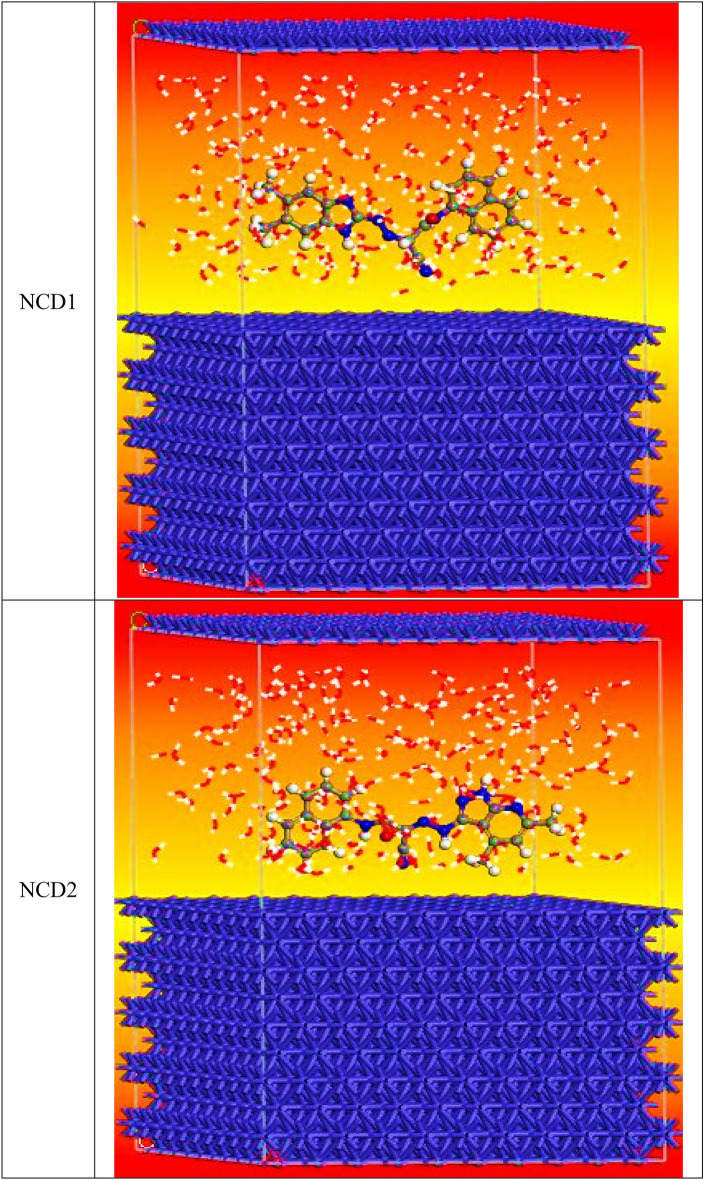
Highest proper adsorption arrangement for the NCDs molecules (final equilibrium configurations) on the iron (110) surface accomplished by an adsorption locator module.

**Figure 17:**
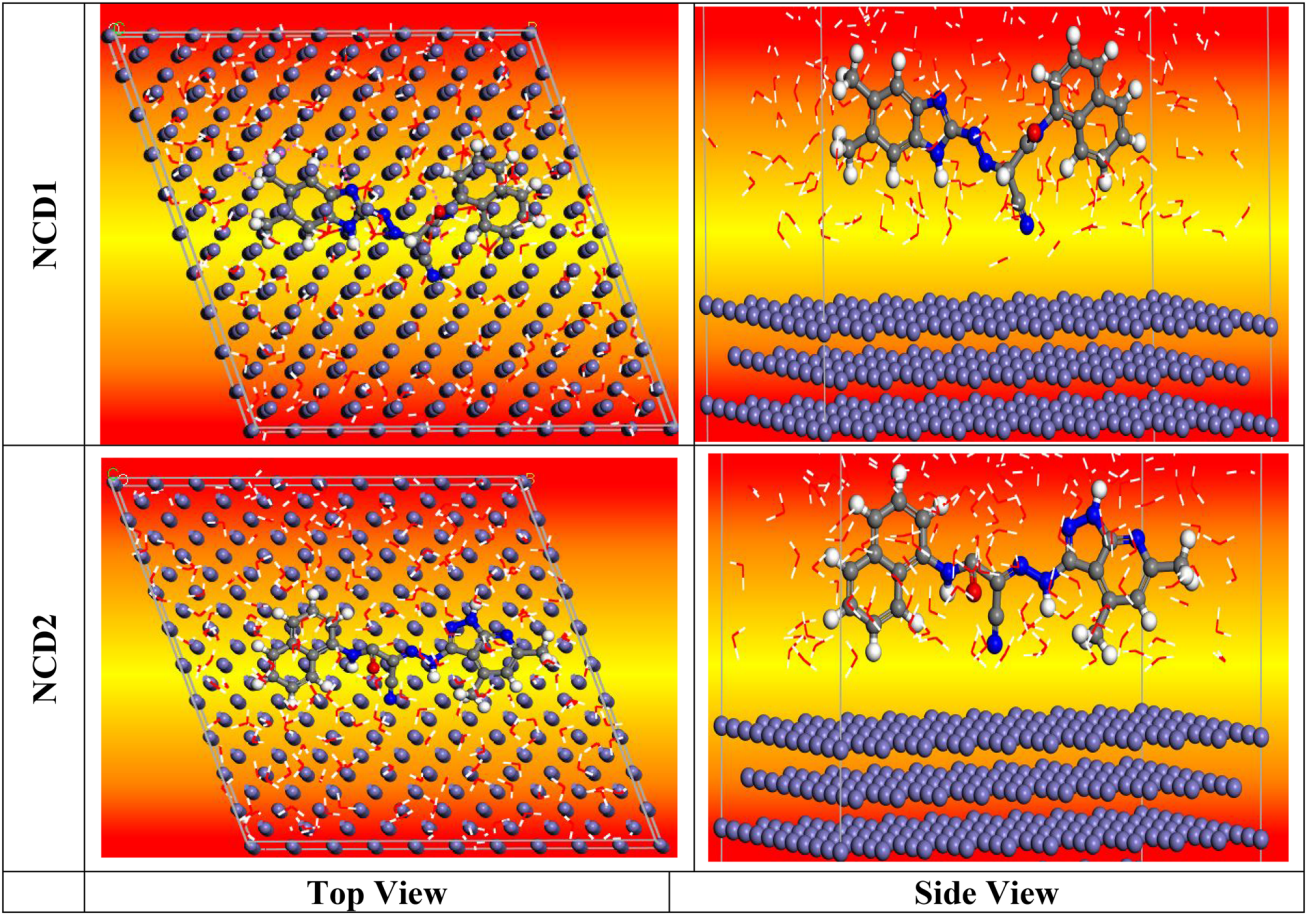
MC side and top views of the ideal configuration for the adsorption of NCDs (**1** and **2**) on Fe (110) surface.

MC is a highly efficient method for identifying the most stable adsorption arrangements of substituted NCDs in an acidic environment. “Fig. 16 displays the Highest proper adsorption arrangement for the NCDs molecules (Final Equilibrium configurations)**,** and Fig. 17 illustrates the most stable perspectives from the lower side and the optimal energy arrangement for the adsorption of NCD (**1** and **2**) on the Fe (110)/50 H_2_O surface. The configurations of the investigated NCDs compounds exhibit a parallel orientation, resulting in maximum contact with the Fe (110) surface. This, in turn, improves the surface coverage. The various parameters obtained from the Monte Carlo simulation are presented in Table 9. The total energy of the substrate-adsorbate contact, expressed in kilojoules per mole (kJ mol^−1^), makes up the parameters. Adsorption energy is the total of the deformation and rigid energies added together. The adsorption energy, expressed in kJ mol^-1^, is the energy released or used during the adsorption of the relaxed adsorbate component onto the substrate surface. The energy of the rigid adsorption, on the other hand, provides the energy, expressed as kJ mol^-1^, that is released (or needed) when the components of the unrelaxed adsorbate are adsorbed on the substrate, that is, before the geometry optimization process. Additionally, the energy released when the adsorbed components of the adsorbate relax on the surface of the substrate is referred to as the deformation energy, measured kJ mol^−1^^77^. dE_ads_/dNi denotes the energy associated with the configurations of adsorbate–substrate interactions, measured in kilojoules per mole. This energy value is obtained by removing one of the adsorbate molecules. Table 9 demonstrates that the adsorption energy of all the inhibitors on the Fe (110) surface is significantly higher than the adsorption energies of H_2_O molecules. This finding implies that the water molecules on the surface of Inconel 800 may be gradually substituted, facilitating the development of a durable layer that can shield the Inconel surface from corrosion. Furthermore, NCD2 exhibits the most negative adsorption energy throughout the simulation process”. The hypothetical simulations are closely aligned with the experimental outcomes.

**Table 9:** MC parameters for adsorption of NCDs on Fe (110).

Structures	Total energy	Adsorption energy	Rigid adsorption energy	Deformation energy	Compound dE_ad_/dNi	H_2_O dE_ad_/dNi
Fe (1 1 0)/(NCD1)/H_2_O	−3123.33	−4023.97	−3948.519	−75.451	−294.787	−9.926
Fe (1 1 0)/(NCD2)/H_2_O	−3130.11	−4028.05	−3950.536	−77.514	−283.578	−11.214

### Inhibition mechanism

**Figure 18:**
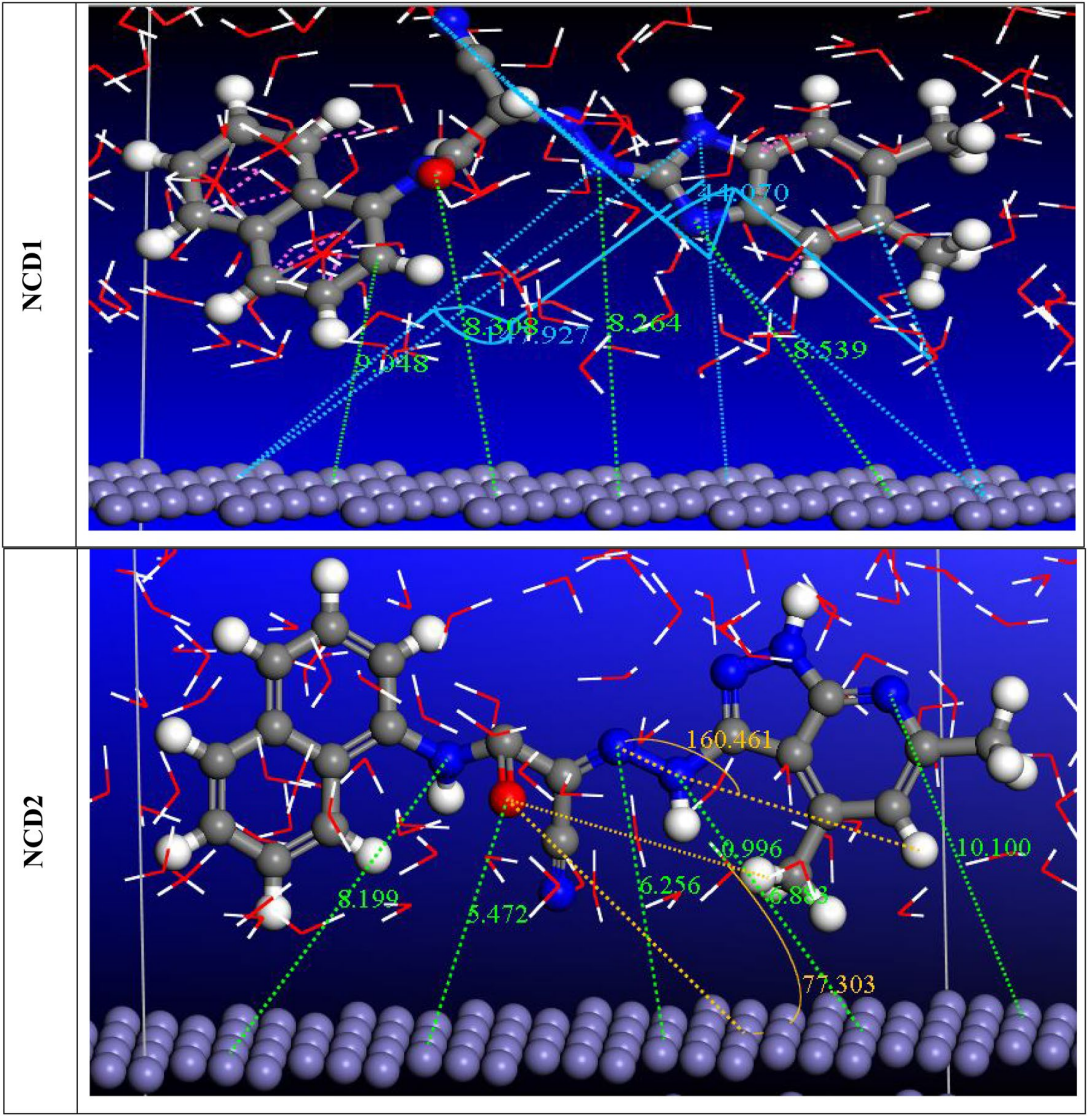
Radial distribution function of the NCDs on the Inconel 800 surface in solution.

**Figure 19:**
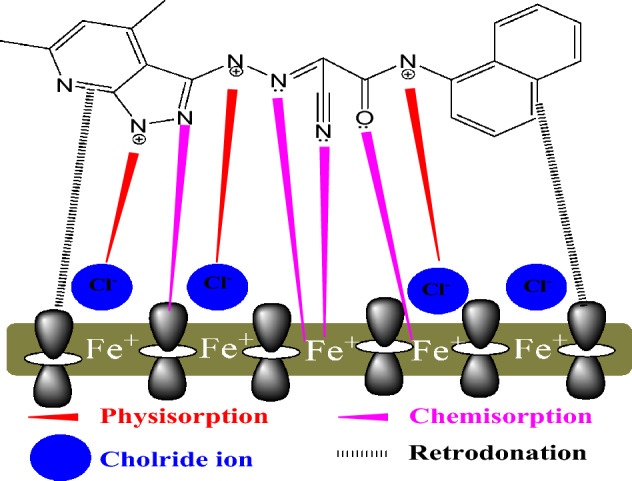
Illustration shape for the adsorption mechanism of NCDs on Inconel 800 surface in HCl solution.

Numerous variables can influence the bonding between the NCD molecules and inhibition degree and Inconel 800 surface. “These variables include the functional groups, the reactivity of the aromatic ring's substituents, the charges on the substrate surface, the electron density, and the corrosive medium's nature^78^. As per the literature, the inhibitor molecule and the Inconel 800 surface interaction could be physical and/or chemically adsorption. The active centers in the adsorption mechanism of the studied inhibitors are identified as the nitrogen atoms and aromatic rings. The inhibition mechanism in this study is interpreted based on the results obtained from both experimental and theoretical analyses, as illustrated in Figs. 18, 19. The experimental and theoretical investigation determined that the following order of reactivity and inhibition efficiency is: NCD2 > NCD1. This discrepancy arises from the dissimilarity in the molecular compositions of the inhibitors. NCD2 contains a greater number of nitrogen atoms in its moiety, which serves as an additional active site and enhances the adsorption of (NCD2) more than (NCD1). The radial distribution function is utilized to estimate the bond length between metal and NCDs. The peak arises from 1 up to 3.5 Å; it is a sign of the length of small bonds, which is associated with chemisorption Fig. 18. In contrast, physisorption is associated with peaks greater than 3.5 Å”^79^. Moreover, the NCDs atom displays that the bond length of iron is higher than 3.5 Å” the inhibitors are physisorption on the iron surface.

NCDs molecules adhere to the Inconel 800 surface through two mechanisms. Firstly, there is an electron transfer from the unshared electron pairs of the aromatic ring's π-electrons to the vacant d-orbital of iron, which has low energy and takes place in the anodic area. Secondly, a chemical coordination bonding occurs between the nitrogen hetero atoms of the NCDs and the iron atoms of the Inconel 800 surface. Furthermore, the inhibition may stem from the process of physical adsorption. The inhibitor molecules become protonated in acidic solutions, resulting in the formation of cationic molecules. These cationic molecules then interact electrostatically with the chloride anions that are adsorbed on the surface of Inconel 800 from the hydrochloric acid solution^80^.

## Conclusions

1. The novel synthesized NCDs acted as an efficient inhibitor for Inconel 800 in 2 M HCl between 25, 30, 35, 40, and 45 °C at all doses. The experimental investigation by electrochemical techniques, a chemical technique, and theoretical studies.
2. The %IE increases with increasing the concentration of NCDs and decreasing the temperature obtaining a higher efficiency of 95.7%, for NCD2 and 91.8% for NCD1.
3. The corrosion process was exothermic and exhibited physical adsorption. The –ve values of ∆G_ads_ indicate the spontaneous adsorption of NCDs on the Inconel 800 surface and the stability of the adsorbed layer on the Inconel 800 surface.
4. The adsorption of NCDs adheres to the Langmuir adsorption isotherm.
5. Analysis employing Tafel plots indicates that the inhibitors adhere to a mixed type.
6. The examination of the morphology of Inconel 800 sheets through SEM, AFM, and FT-IR analysis furnishes valuable insights into their surface characteristics.
7. Density Functional Theory (DFT) calculations are employed to provide a theoretical underpinning for the observed inhibitory behavior of the substances under investigation.

### Supplementary Information

Supplementary Information.

## Data Availability

Data is provided within the manuscript or supplementary information files.
