# Supplementary material for: Electrochemical behavior and theoretical studies of arylazo (1-naphthyl-2-cyanoacetamide) derivatives as new corrosion inhibitors for Inconel 800 in chloride solution
Source: Sci Rep. 2024 Jun 26;14:14683. doi: 10.1038/s41598-024-62795-5 (PMC11199561; doi:10.1038/s41598-024-62795-5)
Supplement: Supplementary file 1 — Supplementary Information. [file 41598_2024_62795_MOESM1_ESM.docx]

Supporting Information

[**Electrochemical Behavior and Theoretical Studies**](https://www.sciencedirect.com/science/article/pii/S0167732221031457) **of Arylazo (1-Naphthyl-2-cyanoacetamide) Derivatives as new Corrosion Inhibitors for Inconel 800 in Chloride Solution**

Mariem. M. Motawea ^1, 2*^

^1^ Department of Chemistry, College of Science, University of Bisha, Bisha 61922, Saudi Arabia

^2^ Delta Higher Institute of Engineering & Technology Mansoura, Egypt

^*^ Corresponding Author E-mail: [dr_mmm_2018@yahoo.com](mailto:dr_mmm_2018@yahoo.com)

**3.1. Synthesized inhibitors (NCDs) [25]:**

***2-Cyano-2-((5,6-dimethyl-1H-benzo[d]imidazol-2-yl) diazenyl)-N (naphthalene-1-yl)acetamide (NCD1):***

Coupling reaction of “1-Naphthyl-2-cyanoacetamide with aromatic amine diazonium salts compound. A cold solution of the appropriate diazonium chloride (2 mmol; prepared by adding cold sodium nitrite solution (0.14 g, 2 mmol) to a cold suspension of the appropriate 5,6-dimethyl-1H-benzo[d]imidazol-2-amine (2 mmol) in conc. HCl (1.5 mL) with stirring) was added, with continuous stirring, to a cold solution of 1 (0.42 g, 2 mmol) at 0–5 ^o^C in pyridine (20 mL). The mixture was left to stand for 2 h, diluted with water, then filtered. The 2-Cyano-2-((5,6-dimethyl-1H-benzo[d]imidazol-2-yl) diazenyl)-N (naphthalene-1-yl)acetamide (NCD1) thus obtained was dried and recrystallized from 2:1 EtOH–DMF”.

***(2-Cyano-N-(naphthalene-1-yl)-2-[(4,6-dimethyl-1H-pyrazolo [3, 4-b] pyridine-3-yl) hydrazono] acetamide (NCD2 ):***

The same procedure used for the preparation of **NCD2** was employed using “4,6-dimethyl-1H-pyrazolo[3,4-b]pyridin-3-amine instead of 5,6-dimethyl-1H-benzo[d]imidazol-2-amine to afford 2-Cyano-N-(naphthalene-1-yl)-2-[(4,6-dimethyl-1H-pyrazolo [3, 4-b] pyridine-3-yl) hydrazono] acetamide”.

**Table S1**: The evaluated Fukui directories NCD1

| C | f+ | ꙍ+ | σ+ | f- | ꙍ- | σ- | Δf | Δꙍ | Δσ |
| --- | --- | --- | --- | --- | --- | --- | --- | --- | --- |
| C1 | 0.002 | 0.042 | 0.004 | 0.030 | 0.628 | 0.060 | -0.028 | -0.586 | -0.056 |
| C2 | -0.009 | -0.188 | -0.018 | 0.002 | 0.042 | 0.004 | -0.011 | -0.230 | -0.022 |
| O3 | 0.014 | 0.293 | 0.028 | 0.057 | 1.192 | 0.113 | -0.043 | -0.900 | -0.086 |
| N4 | 0.007 | 0.146 | 0.014 | 0.021 | 0.439 | 0.042 | -0.014 | -0.293 | -0.028 |
| C5 | 0.002 | 0.042 | 0.004 | 0.061 | 1.276 | 0.121 | -0.059 | -1.234 | -0.117 |
| C6 | 0.005 | 0.105 | 0.010 | 0.049 | 1.025 | 0.098 | -0.044 | -0.920 | -0.088 |
| C7 | 0.002 | 0.042 | 0.004 | 0.047 | 0.983 | 0.094 | -0.045 | -0.941 | -0.090 |
| C8 | 0.006 | 0.126 | 0.012 | 0.072 | 1.506 | 0.143 | -0.066 | -1.381 | -0.131 |
| C9 | 0.001 | 0.021 | 0.002 | 0.012 | 0.251 | 0.024 | -0.011 | -0.230 | -0.022 |
| C10 | 0.003 | 0.063 | 0.006 | 0.064 | 1.339 | 0.127 | -0.061 | -1.276 | -0.121 |
| C11 | 0.002 | 0.042 | 0.004 | 0.043 | 0.900 | 0.086 | -0.041 | -0.858 | -0.082 |
| C12 | 0.003 | 0.063 | 0.006 | 0.044 | 0.920 | 0.088 | -0.041 | -0.858 | -0.082 |
| C13 | 0.002 | 0.042 | 0.004 | 0.063 | 1.318 | 0.125 | -0.061 | -1.276 | -0.121 |
| C14 | 0.003 | 0.063 | 0.006 | 0.019 | 0.397 | 0.038 | -0.016 | -0.335 | -0.032 |
| N15 | 0.13 | 2.720 | 0.259 | 0.001 | 0.021 | 0.002 | 0.129 | 2.699 | 0.257 |
| N16 | 0.136 | 2.845 | 0.271 | 0.007 | 0.146 | 0.014 | 0.129 | 2.699 | 0.257 |
| C17 | 0.026 | 0.544 | 0.052 | 0.001 | 0.021 | 0.002 | 0.025 | 0.523 | 0.050 |
| N18 | 0.048 | 1.004 | 0.096 | 0.002 | 0.042 | 0.004 | 0.046 | 0.962 | 0.092 |
| C19 | 0.035 | 0.732 | 0.070 | 0.002 | 0.042 | 0.004 | 0.033 | 0.690 | 0.066 |
| C20 | 0.025 | 0.523 | 0.050 | 0.001 | 0.021 | 0.002 | 0.024 | 0.502 | 0.048 |
| N21 | 0.103 | 2.155 | 0.205 | 0.005 | 0.105 | 0.010 | 0.098 | 2.050 | 0.195 |
| C22 | 0.042 | 0.879 | 0.084 | 0.002 | 0.042 | 0.004 | 0.040 | 0.837 | 0.080 |
| C23 | 0.024 | 0.502 | 0.048 | 0.001 | 0.021 | 0.002 | 0.023 | 0.481 | 0.046 |
| C24 | 0.034 | 0.711 | 0.068 | 0.002 | 0.042 | 0.004 | 0.032 | 0.669 | 0.064 |
| C25 | 0.035 | 0.732 | 0.070 | 0.002 | 0.042 | 0.004 | 0.033 | 0.690 | 0.066 |
| N29 | 0.052 | 1.088 | 0.103 | 0.022 | 0.460 | 0.044 | 0.030 | 0.628 | 0.060 |

**Table S2**: The evaluated Fukui directories NCD2

| C | f+ | ꙍ+ | σ+ | f- | ꙍ- | σ- | Δf | Δꙍ | Δσ |
| --- | --- | --- | --- | --- | --- | --- | --- | --- | --- |
| C1 | 0.02 | 0.538 | 0.053 | 0.022 | 0.592 | 0.058 | -0.002 | -0.054 | -0.005 |
| C2 | 0.046 | 1.238 | 0.121 | 0.012 | 0.323 | 0.032 | 0.034 | 0.915 | 0.090 |
| O3 | 0.049 | 1.319 | 0.129 | 0.045 | 1.211 | 0.119 | 0.004 | 0.108 | 0.011 |
| N4 | 0.029 | 0.781 | 0.077 | 0.02 | 0.538 | 0.053 | 0.009 | 0.242 | 0.024 |
| N5 | 0.112 | 3.015 | 0.296 | 0.031 | 0.835 | 0.082 | 0.081 | 2.181 | 0.214 |
| N6 | 0.047 | 1.265 | 0.124 | 0.022 | 0.592 | 0.058 | 0.025 | 0.673 | 0.066 |
| C7 | 0.011 | 0.296 | 0.029 | 0.003 | 0.081 | 0.008 | 0.008 | 0.215 | 0.021 |
| C8 | 0.014 | 0.377 | 0.037 | 0.006 | 0.162 | 0.016 | 0.008 | 0.215 | 0.021 |
| C9 | 0.008 | 0.215 | 0.021 | 0.004 | 0.108 | 0.011 | 0.004 | 0.108 | 0.011 |
| C10 | 0.016 | 0.431 | 0.042 | 0.008 | 0.215 | 0.021 | 0.008 | 0.215 | 0.021 |
| C11 | 0.007 | 0.188 | 0.018 | 0.003 | 0.081 | 0.008 | 0.004 | 0.108 | 0.011 |
| N12 | 0.022 | 0.592 | 0.058 | 0.011 | 0.296 | 0.029 | 0.011 | 0.296 | 0.029 |
| N13 | 0.054 | 1.454 | 0.143 | 0.023 | 0.619 | 0.061 | 0.031 | 0.835 | 0.082 |
| N14 | 0.029 | 0.781 | 0.077 | 0.015 | 0.404 | 0.040 | 0.014 | 0.377 | 0.037 |
| C15 | 0.013 | 0.350 | 0.034 | 0.006 | 0.162 | 0.016 | 0.007 | 0.188 | 0.018 |
| C18 | 0.011 | 0.296 | 0.029 | 0.051 | 1.373 | 0.135 | -0.040 | -1.077 | -0.106 |
| C19 | 0.023 | 0.619 | 0.061 | 0.041 | 1.104 | 0.108 | -0.018 | -0.485 | -0.048 |
| C20 | 0.01 | 0.269 | 0.026 | 0.039 | 1.050 | 0.103 | -0.029 | -0.781 | -0.077 |
| C21 | 0.027 | 0.727 | 0.071 | 0.061 | 1.642 | 0.161 | -0.034 | -0.915 | -0.090 |
| C22 | 0.003 | 0.081 | 0.008 | 0.01 | 0.269 | 0.026 | -0.007 | -0.188 | -0.018 |
| C23 | 0.013 | 0.350 | 0.034 | 0.054 | 1.454 | 0.143 | -0.041 | -1.104 | -0.108 |
| C24 | 0.009 | 0.242 | 0.024 | 0.036 | 0.969 | 0.095 | -0.027 | -0.727 | -0.071 |
| C25 | 0.012 | 0.323 | 0.032 | 0.036 | 0.969 | 0.095 | -0.024 | -0.646 | -0.063 |
| C26 | 0.01 | 0.269 | 0.026 | 0.053 | 1.427 | 0.140 | -0.043 | -1.158 | -0.114 |
| C27 | 0.012 | 0.323 | 0.032 | 0.015 | 0.404 | 0.040 | -0.003 | -0.081 | -0.008 |
| C28 | 0.058 | 1.561 | 0.153 | 0.005 | 0.135 | 0.013 | 0.053 | 1.427 | 0.140 |
| N29 | 0.125 | 3.365 | 0.330 | 0.032 | 0.861 | 0.084 | 0.093 | 2.504 | 0.246 |

**Table S3**: Fitting data (slope, intercept & R^2^) were applied to the different adsorption isotherm

| Adsorption isotherm model | Temp.  °C | NCD1 | | | NCD2 | | |
| --- | --- | --- | --- | --- | --- | --- | --- |
|  |  | slope | intercept | R^2^ | slope | intercept | R^2^ |
| Temkin | 25 | 0.34668 | 2.49661 | 0.8563 | 0.30336 | 2.3486 | 0.9362 |
|  | 35 | 0.26789 | 2.06773 | 0.8829 | 0.25105 | 2.02199 | 0.8944 |
|  | 45 | 0.28399 | 2.13378 | 0.9044 | 0.27077 | 2.10737 | 0.8999 |
|  | 55 | 0.26603 | 2.01636 | 0.7516 | 0.26129 | 2.01993 | 0.7745 |
|  | 65 | 0.34204 | 2.32673 | 0.9143 | 0.28745 | 2.13765 | 0.9041 |
| Henry | 25 | 14100 | 0.60212 | 0.9169 | 11400 | 0.7031 | 0.8885 |
|  | 35 | 10850 | 0.60434 | 0.9047 | 10075 | 0.6518 | 0.9131 |
|  | 45 | 11200 | 0.58641 | 0.8895 | 10525 | 0.6339 | 0.8989 |
|  | 55 | 10950 | 0.56083 | 0.8810 | 10800 | 0.5898 | 0.9171 |
|  | 65 | 13675 | 0.46062 | 0.9057 | 11325 | 0.5715 | 0.9080 |
| Freundlich | 25 | 0.19252 | 0.84309 | 0.8927 | 0.15951 | 0.71596 | 0.9385 |
|  | 35 | 0.15678 | 0.64479 | 0.9073 | 0.14013 | 0.58418 | 0.901 |
|  | 45 | 0.17099 | 0.70683 | 0.9038 | 0.15527 | 0.65211 | 0.9399 |
|  | 55 | 0.16399 | 0.65469 | 0.7707 | 0.15502 | 0.62705 | 0.7929 |
|  | 65 | 0.23652 | 0.65469 | 0.8899 | 0.17641 | 0.72559 | 0.9056 |
